# Multivariate Approaches Boosting Lithium‐Mediated Ammonia Electrosynthesis in Different Electrolytes

**DOI:** 10.1002/anie.202416027

**Published:** 2025-01-28

**Authors:** Anna Mangini, Jon Bjarke Valbæk Mygind, Sara Garcia Ballesteros, Alessandro Pedico, Marco Armandi, Ib Chorkendorff, Federico Bella

**Affiliations:** ^1^ Department of Applied Science and Technology Politecnico di Torino Corso Duca degli Abruzzi 24 Torino 10129 Italy; ^2^ Department of Physics Technical University of Denmark Fysikvej Kongens Lyngby 2800 Denmark; ^3^ Istituto Nazionale di Ricerca Metrologica Strada delle Cacce, 91 10135 Torino Italy

**Keywords:** Ammonia, Electrochemistry, Electrolyte, Experimental design, Green chemistry, Lithium-mediated

## Abstract

Ammonia electrosynthesis through the lithium‐mediated approach has recently reached promising results towards high activity and selectivity in aprotic media, reaching high Faradaic efficiency (FE) values and NH_3_ production rates. To fasten the comprehension and optimization of the complex lithium‐mediated nitrogen reduction system, for the first time a multivariate approach is proposed as a powerful tool to reduce the number of experiments in comparison with the classical one‐factor‐at‐a‐time approach. Doehlert design and surface response methodology are employed to optimize the electrolyte composition for a batch autoclaved cell. The method is validated with the common LiBF_4_ salt, and the correlations between the FE and the amount of lithium salt and ethanol as proton donor are elucidated, also discussing their impact on the solid electrolyte interphase (SEI) layer. Moreover, a new fluorinated salt is proposed (i.e., lithium difluoro(oxalate) borate (LiFOB)), taking inspiration from lithium batteries. This salt is chosen to tailor the SEI layer, with the aim of obtaining a bifunctional interfacial layer, both stable and permeable to N_2_, the latter being an essential characteristic for batch systems. The SEI layer composition is confirmed strategic and its tailoring with LiFOB boosts FE values.

## Graphical abstract

A first application of chemometric methods in the field of electrochemical ammonia synthesis: this work explores new salts and electrolyte formulations, studied in a reactor operating under mild conditions and able to provide ≈40 % efficiencies of molecular nitrogen conversion into ammonia.

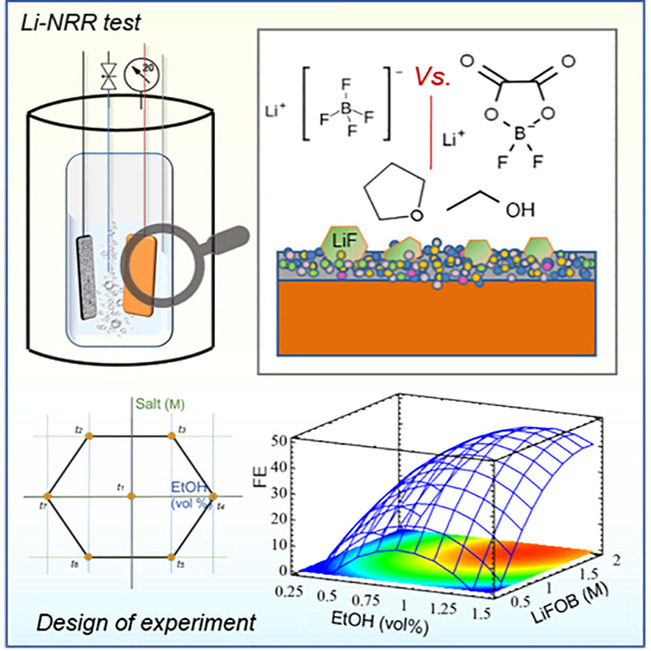

## Introduction

Ammonia (NH_3_) synthesis has recently garnered significant attention not only within the scientific community, but also at political tables.[^1^, ^2^, ^3^] The development of a new strategy to address the energy and climate crisis, alongside the necessity of ensuring fertilizers supply, turns the light on the century‐old Haber–Bosch process, which generates massive carbon dioxide (CO_2_) emissions, i.e. approximately 1.5 t_CO2‐eq_ t_NH3_^–1^ in optimized plants, and high energy consumption.^[4]^ In this context, electrochemical NH_3_ synthesis represents an attractive alternative to the Haber–Bosch process since it enables in situ NH_3_ production,[^5^, ^6^] opening to the decentralization of the process and permitting the use of renewable energy sources.^[7]^ However, to compete with the well‐established Haber–Bosch, research into the electrochemical nitrogen (N_2_) reduction reaction (NRR) for NH_3_ production still has to face significant challenges. A primary obstacle is the competition with the thermodynamically favored hydrogen (H_2_) reduction reaction, which, combined with the low N_2_ solubility in water (H_2_O) and the very stable N≡N triple bond, leads to low Faradaic efficiency (FE) and NH_3_ production rate.[^8^, ^9^] Moreover, this low production implies the critical need for accurate NH_3_ quantification and the assessment of the sources of its production, to avoid misleading results.^[10]^ Recently, the use of aprotic solvents, combined with a lithium salt, as electrolyte has emerged as a promising strategy to meet the values in terms of FE and NH_3_ production rate indicated by the US Department of Energy for the real scalability of the process.^[11]^ In this system, usually referred as “lithium‐mediated” NRR (Li‐NRR), a series of reactions take place almost simultaneously. Initially, Li^+^ ions from the electrolyte are electroplated on the metallic cathode surface forming metallic lithium. There, lithium activates N_2_ to produce intermediate species which are rapidly protonated by a proton donor, e.g. ethanol (EtOH), ultimately forming NH_3_.^[12]^ Upon the release of NH_3_, Li^+^ ions return to the solution restarting the process in a cyclic reaction pathway.^[13]^

Nowadays there are mainly two systems to carry out this process, i.e., the flow and the batch reactors.^[14]^ In the flow cell reactor, N_2_ is supplied through a gas diffusion electrode (GDE) at the cathodic side, the electrolyte is continuously recirculated in the cell, and H^+^ comes from the oxidation of H_2_ at the anode.^[15]^ Those protons are continuously supplied from the anode to the cathode thanks to a shuttle molecule, e.g. EtOH. On the other side, in the single‐compartment batch cell N_2_ is continuously bubbled in the electrolyte or the whole cell is pressurized, e.g. at 20 bar of N_2_, to increase the gas solubility. In this batch case, protons are supplied from a proton donor molecule, e.g. again EtOH, which is in this case depleted during the reaction. At the same time, at the anode the electrolyte is oxidized on an inert electrode, usually a platinum mesh. With this reactor the configuration is simpler, the triple phase boundary at the GDE interphase is not present and there is no flow of gas or electrolyte. Reducing the number of variables affecting the process permits to focus on the N_2_ activation reaction at the cathode.[^12^, ^13^] For these reasons, and despite the disadvantage of decomposing the electrolyte, this setup has been chosen in the present study for the evaluation of the applicability of the design of experiments to the Li‐NRR system.

The secondary reactions ongoing in the Li‐NRR systems are not completely delved, and the current primary interest is that to avoid reactions which could limit the FE and stability of the system. Moreover, lithium presents a particularly low reduction potential (E^0^=–3.04 V vs. standard H_2_ electrode), implying a considerable energy consumption in the plating step, and its reserves are limited, therefore minimizing its losses in secondary products is essential.^[16]^ The electrolyte stability challenge is currently a hot topic in this research field.^[17]^ Indeed, the highly reactive metallic lithium and the wide cell potential imply the risk of overcoming the electrolyte stability window, triggering irreversible reactions. Unavoidably, the freshly plated lithium at the cathode reacts with the electrolyte species, forming a passivating layer, known as the solid electrolyte interphase (SEI).^[18]^ It has been demonstrated that the composition and thickness of the SEI layer are critical for Li‐NRR performances since they influence and could limit the diffusion of the reactants to the cathode surface.^[19]^ An ideal SEI layer should (i) ensure a proper and stable passivation of the plated lithium on the cathode, (ii) facilitate the N_2_ diffusion to the surface, in particular in the batch system, in which N_2_ should cross this layer instead of flowing through the GDE, and (iii) regulate the H^+^ availability to mitigate H_2_ evolution or lithium protonation.^[13]^ The correct reactant ratio at the cathode is essential to exploit the electroplated lithium, avoiding an excessive thickening of the deposited metal, which would adversely affect the FE. Moreover, the electrochemical and mechanical stability of the SEI layer is crucial for guaranteeing long‐term process durability and preventing continuous electrolyte degradation at the cathodic interphase.^[17]^ Therefore, tailoring the formation of the SEI layer is pivotal, and each component of the electrolyte is crucial in shaping the SEI morphology and composition, from the solvent and nature of the lithium salt to additives and proton donor/shuttle molecules.[^20^, ^21^]

In particular, it has been demonstrated that the presence of fluorinated anions capable of being decomposed into lithium fluoride (LiF) at the cathode surface is essential to enhance the FE, guaranteeing a stable SEI layer.^[22]^ Similarly, the presence of EtOH from the outset of lithium deposition has been observed as a crucial element for a proper SEI formation.^[23]^ An in operando study conducted by Deissler et al. has highlighted the dynamic nature of this layer, as well as the need to maintain a sufficiently porous film which allows the proper diffusion of reactants to the cathode surface.^[24]^ The SEI layer, as well as the role of the anion of the salt used, emerged as a critical factor for selectivity and stability also in a recent study of a post‐lithium NRR system, where electrochemical NH_3_ synthesis was mediated by calcium.^[25]^

Despite the great advances made up to date, there is still a significant lack of information regarding the formation mechanism of the SEI layer and its relative composition and influence on the system. Moreover, its reactive and transient nature makes especially tricky the characterizations of a preserved surface.^[24]^ Further in‐depth studies are essential to bridge this knowledge gap and move towards an optimized electrolyte composition for a durable and efficient process.

On the other hand, the integration of modelling and experiments is becoming increasingly popular as a promising alternative to the traditional one‐factor‐at‐a‐time experimental methods, since it allows not only the reduction of the number of experiments to be performed, but also a deeper study of the system. In this context, the design of experiments is an effective tool to gather the maximum amount of relevant data while minimizing cost and time.^[26]^ In brief, the design of experiments involves the use of a set of multivariate mathematical‐statistical tools to study the behavior of a system (or its optimization if that is the purpose) by combining different levels of influencing variables. This combination is organized in a matrix to explore the experimental space efficiently. That way, design of experiments enables cost‐effective data collection and modelling, leading to mathematical functions that describe the studied region. With a low computational cost, the system can be modelled in the whole selected range, overcoming the findings of just local optimum values for the factors, but describing this combinatorial problem with a polynomial equation. By employing these statistical tools in a strictly controlled setup, it is possible to reveal hidden interactions between input factors, as well as their influences on the system, allowing future research to focus on optimizing the most significant factors. The main steps when employing design of experiments and response surface methodology are: selecting the input factors, defining the experimental domain and response variable, conducting experiments, analyzing results, and validating the accuracy of prediction.[^27^, ^28^, ^29^]

In the present study, design of experiments and response surface methodology strategies were applied as the statistical tools to evaluate the correlation between the electrolyte composition and the process performance, discussing the impact of the different input factors, i.e. the salt and EtOH concentrations, not only in the electrolyte environment, but in particular on the SEI layer composition. Given the complexity of Li‐NRR system and the high operational and analytical cost of its study, the design of experiments represents a precious tool that allows to save both time and resources. These techniques have been successfully used in recent works in the lithium batteries field, which shares a similar chemistry, demonstrating to be powerful tools to accelerate system comprehension and optimization.^[26]^

Two different salts were studied. First, the well‐known lithium tetrafluoroborate (LiBF_4_) was selected since it allows us to validate the applicability of this statistical analysis to the Li‐NRR system by comparing with results already presented in the literature.^[17]^ Then, a newly proposed lithium salt, i.e., lithium difluoro(oxalate) borate (LiFOB), was evaluated for Li‐NRR system, as previous studies in lithium batteries showed this compound to decompose into LiF, but also in oxalate groups, resulting in an homogeneous, permeable, and stable SEI layer.[^22^, ^30^] The negative effect of an excess of LiF in the SEI layer in the batch system was also evaluated by testing the application of fluoroethylene carbonate (FEC) as an additive, imitating a strategy used in lithium batteries to enrich the SEI layer with LiF.[^31^, ^32^] The SEI obtained in the batch Li‐NRR system with the two proposed lithium salts (i.e., LiBF_4_ and LiFOB) was initially characterized by means of attenuated total reflection Fourier‐transform infrared spectroscopy (ATR‐FTIR), X‐rays diffraction (XRD), air‐free X‐rays photoelectron spectroscopy (XPS) analysis, and scanning electron microscopy (SEM) analysis. The effect of EtOH content in the electrolyte and its interaction with LiFOB were also deepened with XPS, XRD, and SEM analysis. Finally, the same design of experiments and response surface methodology statistical tools validated with LiBF_4_ allowed to identify, testing only a few experimental points, the optimal electrolyte composition with LiFOB salt.

## Results and Discussion

### FEC Application as an Additive for a LiF‐Enriched SEI Layer

To investigate the role of LiF in the SEI layer, the use of FEC as an additive to enrich the SEI layer with LiF was initially evaluated. FEC 5 wt % was added to a standard Li‐NRR electrolyte (LiBF_4_ 1 M with EtOH 1 vol % in tetrahydrofuran, THF) and tested employing the batch cell system pressurized at 20 bar. As shown in the linear sweep voltammetry (LSV) graph (**Figure S1**), the addition of FEC in the electrolyte resulted in an expected additional reduction peak between 1.0 and 1.4 V vs. Li^+^/Li, ascribed to FEC reduction, as already reported in lithium batteries‐related literature.[^33^, ^34^] However, a detrimental effect on NH_3_ production was obtained with FEC addition. The obtained FE was 2 %, an order of magnitude lower than the FE obtained for the same setup without FEC (ca. 20 %).^[14]^ The test was later repeated at ambient pressure in a glass cell placed into an Ar‐filled glovebox. In that case, a pale‐grey lithium deposit was observed on the copper foil cathode when adding FEC, while without the additive the deposit was dark grey‐black (**Figure S2**). These results suggest a limited N_2_ diffusion through the SEI layer, coherently with the observed increase in stability of metallic lithium in air when covered by an artificial ex situ LiF‐enriched SEI layer.^[35]^ Moreover, Shaofeng et al. calculated that LiF reduced the Li^+^ conductivity in comparison with lithium carbonate (Li_2_CO_3_), LiOH (lithium hydroxide), and lithium hydrogen difluoride (LiHF_2_) at the lithium plating potential.^[22]^

Considering the obtained results, the possibility of using FEC as an additive in the present work was discarded, while the idea of a bifunctional SEI layer enriched with both LiF and organic components seems preferable for the batch Li‐NRR system.

### Investigation of the SEI Layer Composition: a Comparison of LiBF_4_ with LiFOB

XRD and ATR‐FTIR were used to characterize and compare the SEI layers obtained with two different electrolytes, i.e., with LiBF_4_ or LiFOB as salts, on the metallic lithium electrodeposited on the copper cathode, after Li‐NRR tests in the single‐compartment batch glass cell at ambient pressure. Concretely, those analyses aimed at verifying the hypothesis that, when employing the newly proposed salt (i.e., LiFOB), a less fluorinated SEI layer was formed with respect to the LiBF_4_ case.

**Figure 1:**
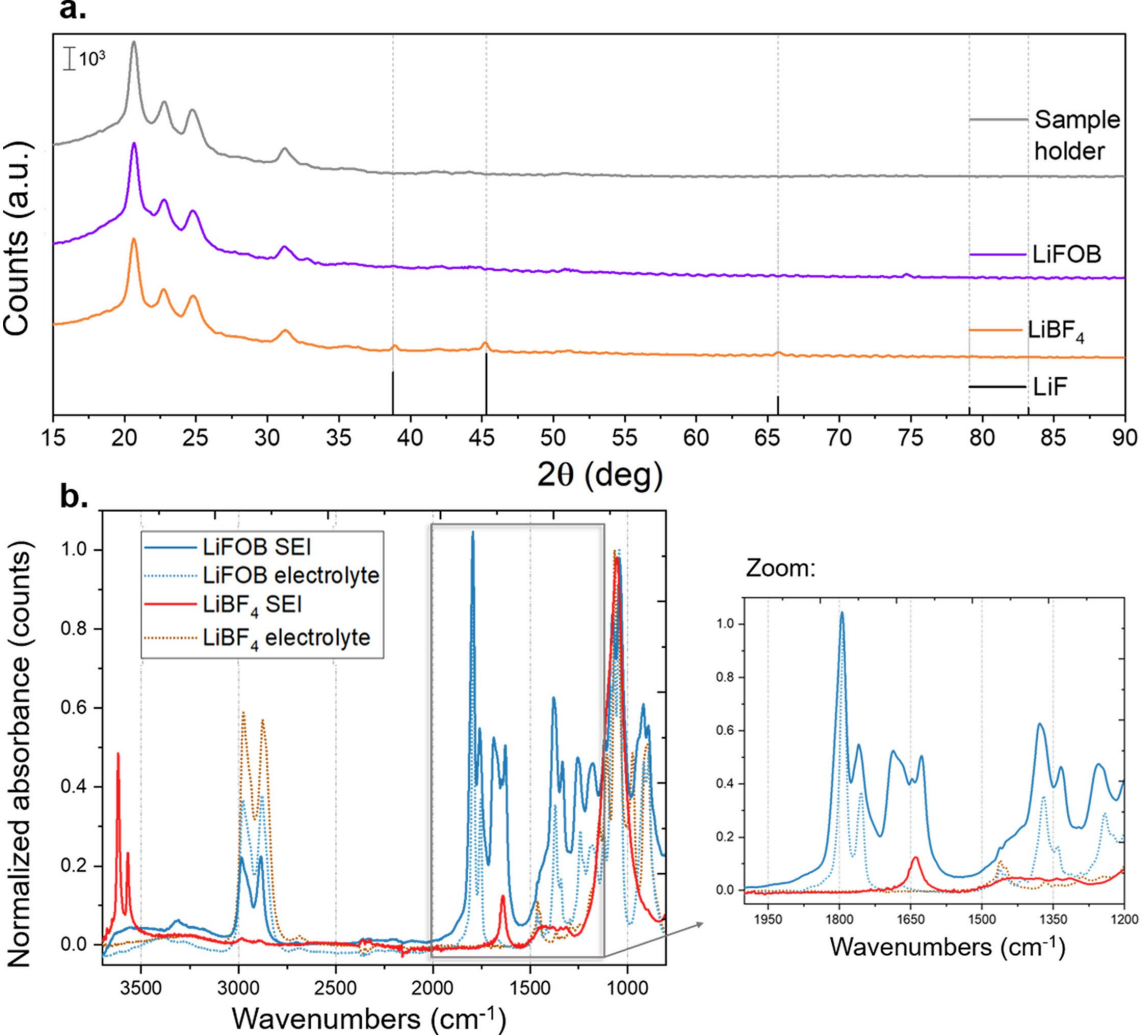
**a**. Air‐free XRD patterns of deposited lithium with SEI layer obtained on the copper cathode after a Li‐NRR test at ambient pressure, with LiBF_4_ 1 M (orange line) or LiFOB 1 M (purple line) and EtOH 1 vol % in THF as electrolyte. The grey line is the pattern of the air‐tight polyether ether ketone (PEEK) dome sample holder. **b**. ATR‐FTIR spectra of the cathode surface with the SEI layer, obtained after a Li‐NRR test at ambient pressure. The orange line is the spectra of the sample obtained with LiBF_4_ 1.25 M and EtOH 0.88 vol % in THF as the electrolyte, the light blue line corresponds to the sample obtained with LiFOB 1.25 M and EtOH 0.88 vol % in THF. The dashed lines are the spectra of the respective electrolytes before the test. The absorbances were normalized by dividing for the maximum of each curve.

Air‐free XRD analysis of the lithium deposit covered by the SEI layer (Figure 1a) clearly shows LiF peaks when LiBF_4_ was used,[^17^, ^22^] while for LiFOB this component was not observed in the spectra. Even if the presence of LiF was expected as consequence of LiFOB decomposition,^[36]^ the amount and crystallinity of LiF particles obtained with LiFOB is expected to be lower than the ones obtained with LiBF_4_, and thereby not detectable through this analysis.

The different SEI layer compositions obtained for the two studied salts were also confirmed by the ATR‐FTIR spectra of the lithium deposit covered by the SEI layer (Figure 1b). When LiFOB was used, additional peaks at 1625 and 1675 cm^–1^ were detected; such peaks were not present in the spectra of the fresh electrolyte before the Li‐NRR experiment (i.e., spectra used as blank). These peaks could be related to C=C or to C=O groups.^[37]^ The latter supports the presence of lithium oxalate (Li_2_C_2_O_4_) in the SEI layer, as previously observed also in the SEI layer in lithium batteries with lithium bis(oxalate) borate (LiBOB) salt in the electrolyte.^[38]^ When LiBF_4_ was employed, two sharp peaks at 3615 and 3561 cm^–1^ were observed in the spectra of the analyzed sample, and they could correspond to the *ν*_3_ (symmetric stretch) and *ν*_1_ (asymmetric stretch) modes of H_2_O molecules adsorbed onto some Lewis sites, as also confirmed by the additional bending band at 1635 cm^–1^.^[39]^ These unexpected peaks suggest the presence of absorbed H_2_O on the SEI layer obtained with LiBF_4_, and thus higher hydrophilicity of this layer compared to the one formed with LiFOB.

The presence of additional C=O groups in the SEI layer when LiFOB is used was further supported by air‐free XPS with depth profiling, as presented in the following section.

### Study of the SEI Layer Composition Obtained with LiFOB Salt and Different EtOH Amounts

To confirm and deepen the effect that changes in the molar ratio between the proton donor and the salt could have on the SEI layer composition, further characterizations, i.e., air‐free XPS, SEM, and XRD analysis were performed on lithium‐deposited samples obtained with LiFOB and with three different amounts of EtOH (i.e., 0, 0.88, and 1.5 vol %).

**Figure 2:**
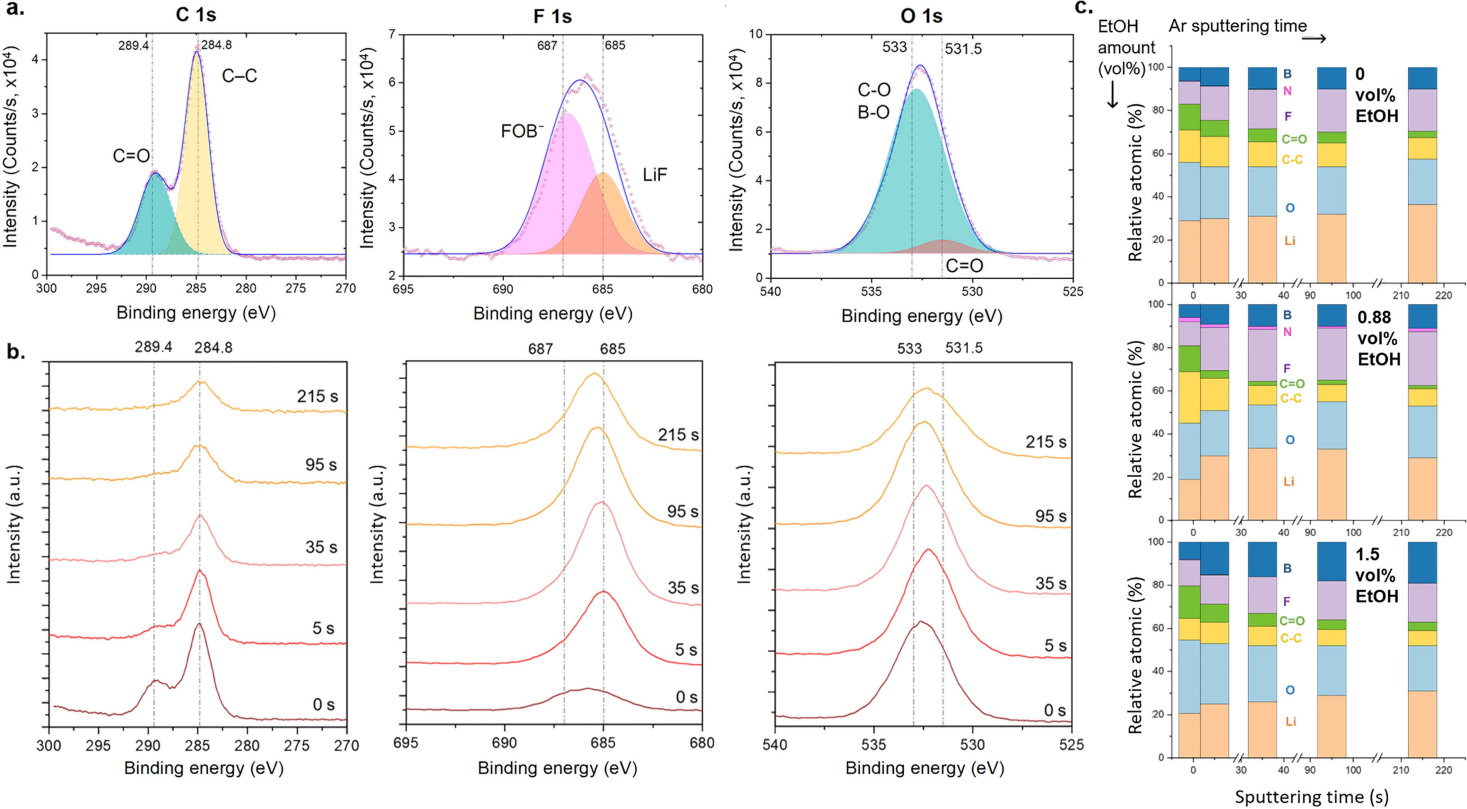
**a**. Air‐free XPS elemental spectra of C 1s, F 1s, and O 1s, with peak fitting, and **b**. the respective depth profiles at different Ar sputtering times. The blue line in the peak fitting (**a**.) corresponds to the cumulative peak fit, while the red dots are the raw data. **c**. Relative atomic % for different elements (related to different colors), compared at different Ar sputtering time. The three stacked graphs correspond to different samples with different EtOH vol%, i.e., from the top to the bottom 0, 0.88, and 1.5 vol %. The presented data are resumed in **Table S1**. All the samples were obtained after a Li‐NRR test in the not pressurized glass cell, with LiFOB 1.25 M and EtOH 0.88 vol % in THF or different EtOH amount as specified.

Air‐free XPS experiments performed on cathode samples after test with LiFOB 1.25 M and EtOH 0.88 vol % in THF as electrolyte (Figure 2 and **Figure S3**) are discussed.

The F 1s spectrum shows a peak which could be deconvoluted into two peaks, the first centered at 685 eV, attributed to LiF, and a further peak at 687 eV, traceable with B−F species coming from the anion of the lithium salt.^[40]^ The latter is predominant in the surface layer and decreases in comparison to the LiF‐corresponding peak at higher sputtering time; the same trend has been reported in literature for LiBF_4_.^[22]^ This finding supports the suggested formation of LiF particles in the LiFOB‐obtained SEI layer, even if not detected in the air‐free XRD (Figure 1a).

Coherently, the B 1s spectrum shows a predominant peak at about 194 eV on the surface, attributable again to the salt anion B−F binding.^[40]^ At higher sputtering time, the peak is shifted towards lower binding energy, suggesting the presence of oxidized species or Li–B bonds, e.g. a binding energy of 192 eV could be attributed to BO_x_ and the value 192.5 eV could be related to Li_2_B_4_O_7_ species. Accordingly, the Li 1s spectrum presents a peak which could be deconvoluted in a main peak at about 56 eV, referable to LiF, and a further smaller peak at 55.4 eV relatable to Li_2_CO_3_ or Li_2_C_2_O_4_. At higher sputtering times, the shifting of the peak at higher binding energy could be related to the additional presence of the metallic lithium at 57.5 eV. The spectra of O 1s also presents a broad peak which could be deconvoluted in two, i.e., a peak at about 531.5 eV attributable to metal carbonates or organic C=O bonds, and a further peak at 533 eV relatable to C−O bonds or to B−O bonds.^[41]^ The presence of C=O groups in the SEI layer, coming for example from the expected species of Li_2_C_2_O_4_, was supported also by the peak at 289.4 eV in the C 1s spectrum, again relatable to C=O bond.^[41]^ The presence of this element, in particular in the outer layer, was coherent with the suggested decomposition of LiFOB at the cathodic interface. Moreover, the presence of this additional peak at higher binding energy has not been observed in literature for the air‐free XPS C 1s spectra obtained when LiBF_4_ is used in the electrolyte.^[22]^ As supposed, these findings suggest a mixed bifunctional interface, in which LiF is not a predominant element of the passivation film as it is in LiBF_4_ case.

For the N 1s spectra, a peak at 399 eV attributed to organic nitrogen species was observed, similar to the peak registered for LiBF_4_.^[22]^ With depth profiling, this peak is shifted to 400.5 eV, which could be attributed again to organic nitrogen species, e.g. to C–NH_2_. However, the presence of metal nitrides, usually observed at about 397 eV, was not detected.

Moreover, the peak of Cu 2p is predominant in the survey after 215 s of sputtering (**Figure S4**), suggesting that, at Ar sputtering time higher than the registered ones, the composition of the SEI layer should not present further changes. With depth profiling (**Figure S5**), the C relative atomic % decreased, while Li, F, and B increased their relative amount. This observation is coherent with a typical lithium battery SEI layer, usually resulting in a mosaic structure, with a stable but permeable inorganic inner layer covered by a more flexible interphase.^[42]^ Moreover, a similar dual SEI layer composition has been observed from in situ neutron reflectometry operated on a similar Li‐NRR system using LiBF_4_.^[43]^

From the depth profiles in Figure 2c and **Figure S6**, some differences emerged when different vol% of EtOH were used. The decrease of the relative atomic % of C, and the simultaneous increase of F and B with depth profiling, is evident for all samples, corroborating the typical trend of a bi‐layer SEI.[^42^, ^43^]

As expected, the amount of deposited lithium is considerably higher without EtOH: due to proton donor scarcity, the Li‐NRR process is limited and the electric current imposition leads to lithium accumulation. On the contrary, Cu 2p peaks are visible after only 95 s of Ar sputtering time in the survey spectra of the sample with more EtOH (**Figure S4**), suggesting a thinner plated lithium layer deposit. The C 1s peak at higher binding energy (i.e., 289.4 eV), previously related to C=O bond, was observed for all samples, supporting the presence of oxalate species in the SEI layer coming from LiFOB (**Figure S7**). This peak decreases with the depth profile in all cases, even if, for the sample with the higher amount (i.e., EtOH 1.5 vol %), it presents a relative amount similar to the other C 1s peak at 284.8 eV, while B and O relative atomic % overcome the F relative amount (**Figure S6**).

To further investigate the SEI layer composition of samples with different EtOH vol% in the electrolyte (i.e., 0.88 and 1.5 vol %), XRD analysis was performed (**Figure S8**). The obtained patterns corroborate the hypothesis of the formation of oxalate groups in the SEI layer, showing peaks ascribable to Li_2_C_2_O_4_, and the sample obtained with more EtOH in the electrolyte also shows additional peaks attributable to Li_2_CO_3_.

**Figure 3:**
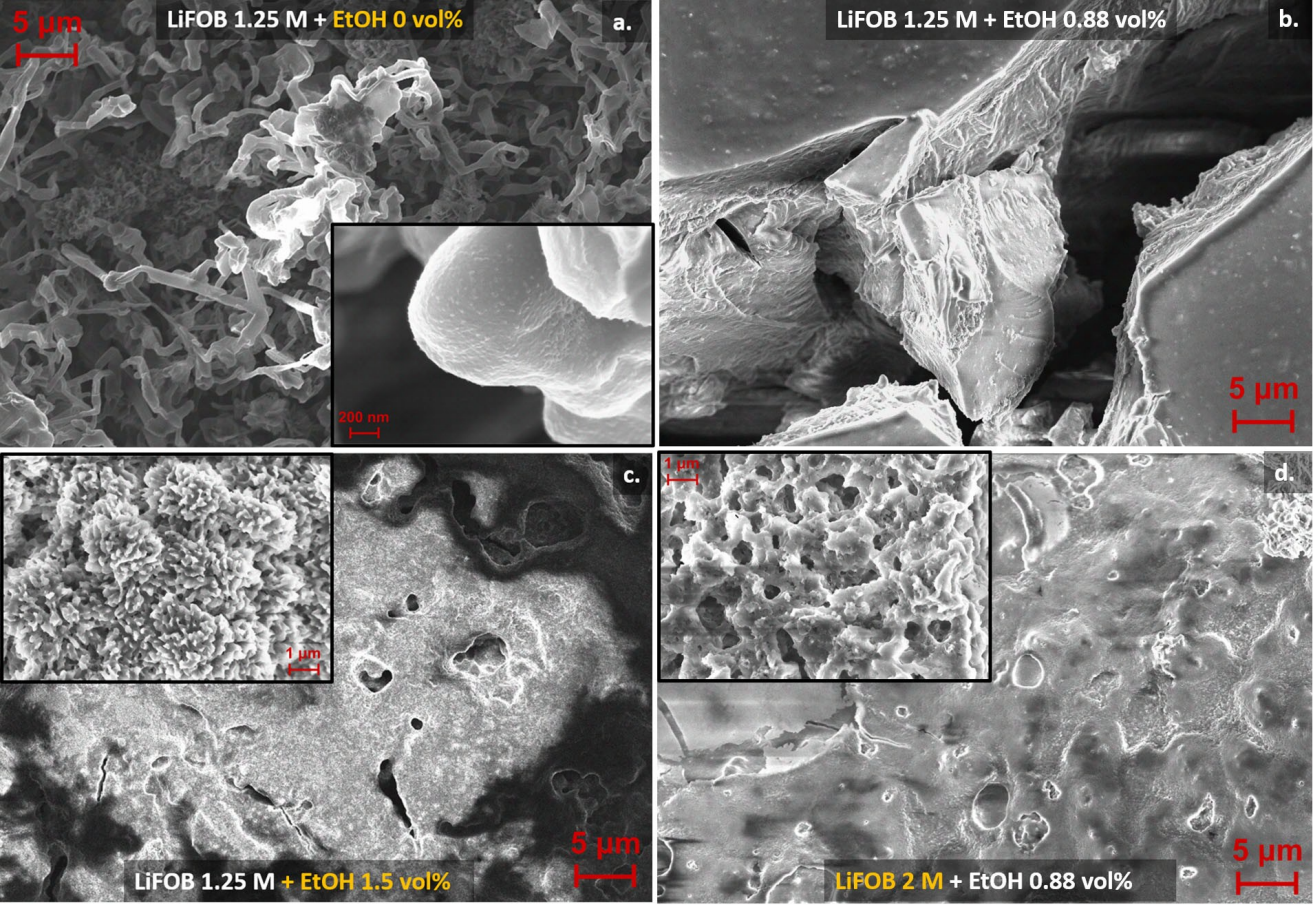
SEM images of the of deposited lithium with SEI layer obtained on the copper cathode after a Li‐NRR test at ambient pressure, with an electrolyte composed of: **a**. LiFOB 1.25 M in THF, **b**. LiFOB 1.25 M with EtOH 0.88 vol % in THF, **c**. LiFOB 1.25 M with EtOH 1.5 vol % in THF, and **d**. LiFOB 2 M with EtOH 0.88 vol % in THF. The insets in **a**., **c**., and **d**. show the images of the corresponding samples at higher magnification.

Finally, SEM measurements were carried out to observe changes in the SEI layer morphology when adopting different EtOH and salt concentrations. SEM measurements were performed minimizing the air exposure of the samples, as described in the Experimental Section. The results, presented in Figure 3 and in **Figures S9–S10**, at lower and higher magnification, respectively, elucidated the high variability of the formed SEI layer with different electrolyte compositions. Without EtOH (Figure 3 **.a**), the obtained SEI layer showed a filamentous morphology, driven by the dendritic electrodeposition of lithium. This result was in line with the SEI layer obtained on a copper electrode in a similar setup, when LiBF_4_ 1 M in THF was used as electrolyte.^[18]^ In that case, the cryo‐TEM analysis showed the metallic lithium lattice presence behind the mosaic SEI layer.^[18]^ Moreover, the presence of nanometric particles could be observed on the deposit surface in Figure 3 **.a** and in its higher magnification (**Figure S10.a**), coherently with the hypothesized LiFOB decomposition in capped LiF particles, as following detailed.^[36]^ When EtOH 0.88 vol % was added to the electrolyte (Figure 3 **.b**), the formation of a dual layer was evident: the surface appeared flatter and more homogeneous, but characterized by open cracks, which revealed a collapsed structure behind the outer layer. This finding aligns with a dual‐composition SEI layer, as observed from XPS analysis. At a higher EtOH amount, i.e., 1.5 vol %, (Figure 3 **.c**), the surface resulted in a porous and open structure, characterized by numerous open channels and cavities of hierarchical porosity. It should be noted that the deposit was less stable on the electrode in this case, and only a thin film was observed after the test, coherently with the XPS analysis. When the salt molarity was increased from 1.25 M to 2 M, with 0.88 vol % EtOH (Figure 3 **.d**), instead, the morphology resulted dense and irregular, and characterized by sub‐micrometrical particles and flakes. This sample was the more similar to the result previously presented with LiBF_4_ 1 M and EtOH 1 vol %.^[18]^ In similarity to that study, only the sample without EtOH presented a continuous deposit, while the others resulted in an inhomogeneous coverage of the copper foil and were characterized by an increased electrical insulation of the surface.

### Discussion on the Differences Observed in the SEI Layer: Trying to Guess Correlations with the Electrolyte Composition

At this point, the correlation between the molar ratio of proton donor and lithium salt in the electrolyte with the SEI layer composition did not show a clear trend. The performed characterization was considered insufficient for a clear comprehension of the competition towards decomposition between the different species in the electrolyte at the cathodic interface, and the consequent SEI layer formation.

In theory, the great influence that the distribution of the species in the electrolyte in the proximity of the working electrode has on the reaction rate and selectivity is well known. Indeed, when the electrode potential overcomes the reduction potential of more than one component in the electrolyte, both the nature and the configuration of the species near the cathode determine which species will be more prone to reduction.^[44]^

Considering the remarkably low applied potential used in Li‐NRR tests, all the compounds, except for THF, could be reduced at the cathode, as their reduction potentials are surpassed (**Table S4**). The applied potential must indeed be equal or lower than 0 V *vs* Li^+^/Li, as it is necessary to overcome the lithium electroreduction potential and facilitate its plating. Moreover, even THF may participate to the SEI layer formation, reacting chemically with electroplated lithium.^[45]^ Consequently, the distribution of species facing the electrode will determine the composition and morphology of the SEI layer.

As in the theoretical model for batteries, also in the Li‐NRR system the charged ions may displace in the electrolyte with different configurations, depending on the solvent and additive nature and solvation ability.[^46^, ^47^, ^48^, ^49^] The Gutmann donor number is a useful parameter for predicting the ions configuration, as it measures the ability of an uncharged molecule to donate electron pairs to the cation, and thus serving as a descriptor of the solvation ability.^[50]^ The moderately polar THF, typically used in Li‐NRR studies,^[51]^ has a relatively low donor number (20 kcal mol^−1^), leading to a predominant distribution of contact‐ion‐pairs.^[52]^ Based on these considerations, the SEI layer was expected to be primarily influenced by the nature of the anion.[^22^, ^46^]

Regarding LiBF_4_, it has been observed to form a SEI layer with a significant amount of LiF, simultaneously avoiding an excessive degradation of the salt to HF in the presence of H_2_O.[^24^, ^53^, ^54^]

The organic and fluorinated anion of LiFOB, on the other hand, recently demonstrated promising performances in the field of lithium batteries,[^55^, ^56^] even at very low concentrations.^[57]^ The role of the SEI layer in this context has also been extensively investigated.^[58]^ Notably, compared to other lithium salts proposed for Li‐NRR, such as lithium bis(trifluoromethanesulfonyl)imide (LiTFSI) or lithium triflate (LiCF_3_SO_3_),^[59]^ LiFOB offers the advantage of avoiding sulfonic species, which have been shown to deactivate the PtAu anode catalyst during the H_2_ oxidation reaction.^[60]^

The SEI layer formed with LiFOB in lithium batteries appeared to mirror the characteristics of an ideal SEI for Li‐NRR in batch systems. In particular, the decomposition of LiFOB leads to a SEI layer with both LiF and oligomeric species with C=O groups, Li_2_C_2_O_4_, and boron oxalates simultaneously.^[30]^ Furthermore, the morphology of the SEI layer resulting from LiFOB decomposition has been demonstrated to be remarkably homogeneous and compact, featuring nanometric, uniformly distributed LiF particles encapsulated within a thin film. This capping ability of the oxalate species has been correlated to a consequent uniform potential distribution at the interface, conferring stability and enhancing the device performance.[^36^, ^37^] This dual functionality of the SEI layer obtained by LiFOB decomposition, being both uniformly permeable, and stable, qualifies LiFOB as a bifunctional salt.^[41]^ Moreover, this salt has demonstrated remarkable resistance to reactivity with H_2_O impurities in batteries, effectively reducing its self‐decomposition into HF.^[57]^

The SEI layer obtained in this study with LiFOB was found, based on the reported characterization, to align with previous literature in both composition and morphology. Applying LiFOB in a batch cell for Li‐NRR was expected to increase the SEI layer uniformity and the N_2_ affinity at the electrode surface. The need for a bi‐functional SEI layer in Li‐NRR was further supported by the result observed upon the addition of FEC to the system, which led to a significant decrease in performance. The addition of FEC to the electrolyte has previously been reported to increase the number of contact‐ion‐pair, coordinating Li^+^ and favoring FEC reduction.[^31^, ^52^] The reduction of FEC at the interface has been supposed to follow a de‐fluorination or ring‐opening reaction, resulting in the formation of LiF and poly‐vinyl carbonate or −CHF−OCO_2_‐type compounds.^[61]^ Consequently, this additive is usually applied in batteries to create a LiF‐rich SEI layer with enhanced mechanical and electrochemical stability.^[34]^ However, an excess of LiF in Li‐NRR system was supposed to limit the N_2_ diffusion, as schematically presented in **Figure S11**. On the other side, it has already been demonstrated that introducing a limited amount of oxygen in the feed gas to the cell increases the FE.^[62]^

The combination of LiFOB with EtOH, in the Li‐NRR system, was observed to further modify the SEI layer. Small variations in the relative amounts of salt and EtOH resulted in significant changes in both the morphology and composition of the SEI layer. The electrolyte configuration could be different in each electrolyte composition,^[49]^ and the reduction potential of LiFOB (i.e., 1.6 V *vs* Li^+^/Li), together with the presence of double bonds in its structure, suggest that this molecule can undergo into continuous decomposition.[^60^, ^63^] However, as observed in lithium metal batteries, the proper balancing of different compounds could stabilize the system, leading to the formation of a stable and passivating SEI layer that effectively limited salt reduction.^[58]^ Indeed, as reported, the SEI layer usually shows a complex mosaic‐structure, with an inner layer characterized by a higher amount of inorganic species, rapidly formed on the freshly plated lithium due to the salt anion chemical or electrochemical decomposition at the interface, covered by a more flexible and usually organic interphase, in‐line with the observed multilayered structure in the SEM analysis.^[42]^

Given the unpredictable behavior of various electrolyte components and the resulting SEI layer composition, the multivariate approach and the design of experiments could be a powerful tool for identifying the optimal composition and uncovering hidden correlations between these components.

### Design of Experiments Application towards the Electrolyte Composition Optimization

#### Variables Evaluation and Setting

**Figure 4:**
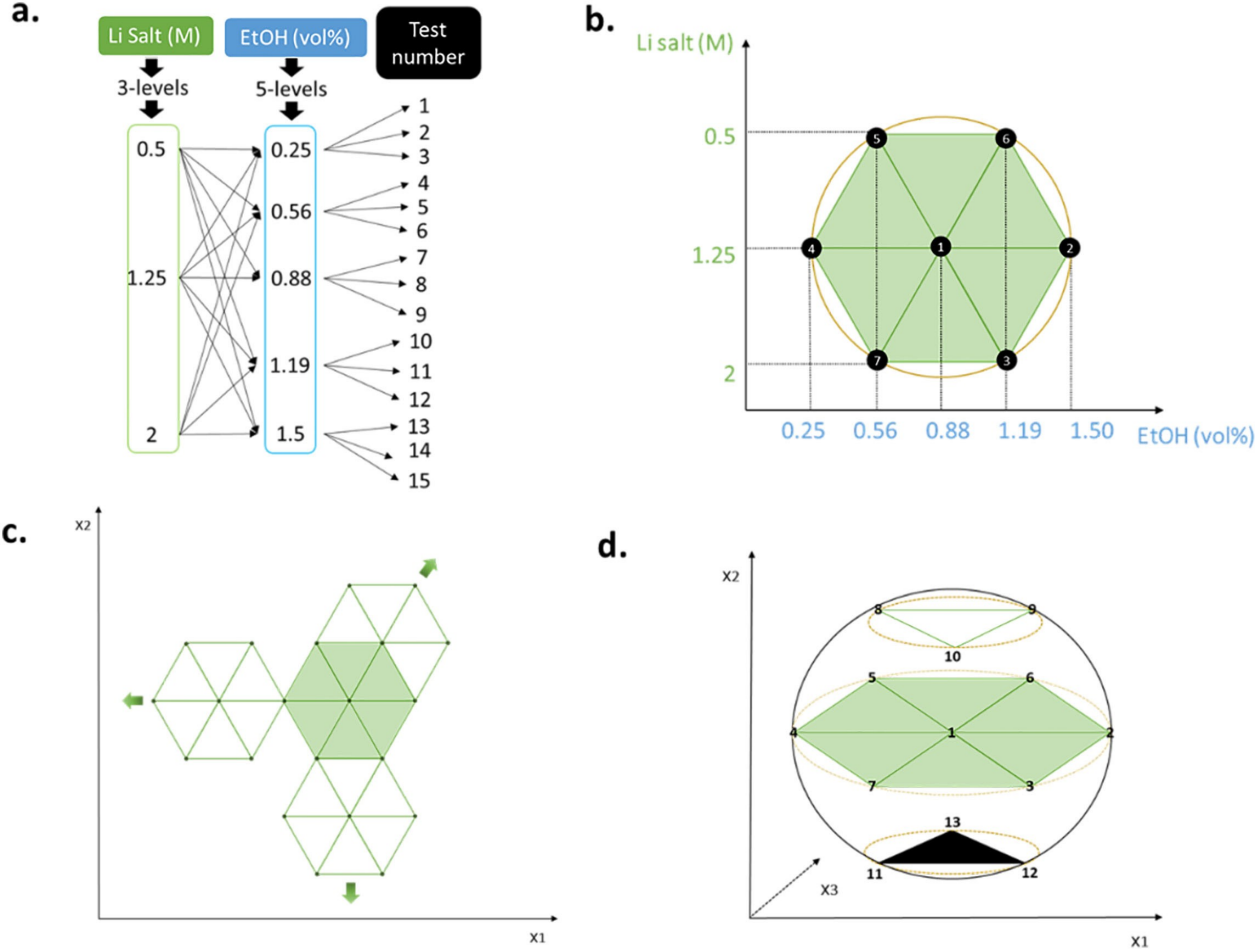
Schematic representation of the same experimental domain for **a**. the one‐factor‐at‐a‐time methodology and **b**. a two‐factor Doehlert matrix; **c**. examples of displacement possibilities of the experimental domain from an original two‐factor Doehlert design, and **d**. spatial distribution of a two‐factor experimental design (points 1–7) expanded to a three‐factor design (points 1–13).

Design of experiments was selected since, as already stated in the introduction section, and depicted in Figure 4 **.a** and **4.b**, it allows process modelling with a smaller number of trials. Concretely, a two‐factor Doehlert matrix was employed, and results were analyzed and modelled employing response surface methodology. The Doehlert matrix was selected since it offers several advantages, i.e. it provides the possibility of moving the experimental region while taking advantage of points already explored in the previous design (Figure 4 **.c**), it allows future expansion of the experimental domain by adding other input factors (Figure 4 **.d**), and it enables assigning different levels to each factor, allowing, for example, a deeper study of factors that may have a greater influence on the system.

The concentration of the lithium salt and the EtOH vol% in the electrolyte were chosen as input factors and the FE as the response variable. Their influence on the SEI layer was then indirectly measured, evaluating the efficacy of this layer by the ammonia produced in the system.

The concentration of the salt in the electrolyte (i.e., the first input factor selected) was chosen as it has a direct influence on the solvation shell, increasing the number of contact ion pairs and the electrolyte viscosity. Those changes, also reflected in the mobility of ions in the electrolyte, affect the rates of electrochemical reactions, including the formation of the SEI layer.[^20^, ^46^, ^64^] The effect of molarity can be dual, as ultra‐low‐concentrated electrolytes have recently been reported to achieve performance comparable to that of highly concentrated electrolytes in batteries.[^57^, ^65^]

With that in mind, the salt molarity was varied between 0.5 and 2.0 M. These values were selected considering previous literature results, as the increase of the FE with molarity has already been proved for different salts.[^22^, ^66^] Even if a further increase in molarity could be beneficial for the FE, 2 M was selected as a maximum value mainly due to (i) solubility issues, in particular considering the need to move towards a more stable, safer, and sustainable solvents than THF (considered cancerogenic),^[17]^ (ii) the intention to avoid an increase in the electrolyte dynamic viscosity, which could be detrimental due to the mass‐transport,^[59]^ and (iii) the fact that higher molarity would mean a higher cost.

EtOH concentration, set as the second factor, was studied in the range of 0.25 vol % ‐ 1.5 vol %. These values were selected with the motivation of including a fairly low minimum, still able to protonate nitrogen, and a moderately high value, which would overcome the expected optimal concentration for LiBF_4_ in the same setup, i.e., 1 vol %.^[22]^ A thresholding behavior for proton donors as a function of their concentration has been observed in numerous studies.[^15^, ^20^, ^51^] This phenomenon has been linked to the competition among various EtOH‐related reactions and its role in SEI layer formation.^[19]^ Indeed, in addition to donating proton for NH_3_ synthesis, EtOH has been observed to decompose into lithium ethoxide (LiEtO), which was observed to be essential for the system, as it is believed to favor N_2_ diffusion through the SEI layer. However, EtOH can also have two negative effects: (i) shifting the selectivity towards hydrogen evolution at the expense of ammonia synthesis and (ii) generate lithium hydride (LiH), an undesired secondary product.[^23^, ^24^] For those reasons, the EtOH concentration was assessed in the design of experiments with a higher level of detail, setting 5 levels for this factor, while only 3 levels were adopted for the lithium salt molarity.

FE was chosen as the response variable since it is nowadays the main performance evaluator parameter. However, the NH_3_ production rate and the system stability should also be considered as fundamental performance parameters of the process.^[67]^ To this aim, in the present study, the production rate was indirectly evaluated by keeping constant the reaction time and electrode area, resulting in a value directly proportional to the FE for each experiment. Finally, even though energy efficiency is recognized as a mandatory parameter to asses Li‐NRR sustainability in comparison with the Haber–Bosch process, in this study it was not calculated due to the low technology readiness level of the applied system, and the consequent lack of data and information for a proper comparison with other systems. Indeed, performing a standardized calculation with defined boundaries is still a tricky issue in the field.^[16]^

#### Setup and Experimental Conditions Setting

A crucial step when employing design of experiments is to select a stable system that allows the rest of the factors affecting the process to remain as constant as possible, minimizing error and obtaining a meaningful and reliable model capable of explaining the variability of the response based on the variation of the studied input factors.

The batch glass cell, pressurized at 20 bar, was chosen as the setup to critically focus on the cathodic reactions, limiting perturbations on the system with unaccounted factors, e.g. the mechanical stress of the gas and liquid flows at the triple phase boundary on the deposited lithium and the corresponding SEI layer. It should be noted that in this setup the expected anodic reaction is the electrolyte oxidation, resulting in a setup not suitable for long‐term stability test.^[23]^

#### Fixed‐Parameter Setting: Evaluating the H_2_O Content Effect

The presence of impurities could also affect the electrolyte species configuration and degradation, and among them H_2_O has a major effect depending on its concentration. A higher H_2_O content has been widely discussed to prompt competing reactions as hydrogen evolution and the irreversible conversion of metallic lithium to LiOH, inhibiting N_2_ activation.[^17^, ^68^, ^69^] In fact, a concentration of 1800 ppm has been reported to suppress the Li‐NRR reaction in the batch system completely.^[70]^ Even though, a certain tolerance to an amount of H_2_O of approximately 300 ppm has also been reported for the flow‐cell setup,^[17]^ while a value of 400 ppm showed a drastic drop of the FE.

Considering that experiments in the autoclaved cell were conducted outside a glovebox, adventitious differences in the autoclave sealing time entail the risk of increasing the H_2_O content by hundreds of ppm. To ensure precise statistical calculations, to keep the effect of this factor as constant as possible, is vital. For that purpose, a series of preliminary tests were performed to carefully evaluate this parameter. The electrolyte composition of LiBF_4_ 1 M in THF with EtOH 1 vol % was firstly tested in the autoclave with 200, 1000, or 2000 ppm of H_2_O. The registered FE (**Table S5**) showed a slight decrease in performance between 200 and 1000 ppm, while at 2000 ppm the effect of H_2_O was detrimental and no NH_3_ was detected. Then, the range of H_2_O content was narrowed, and the electrolyte composition at the central point of the experimental region, i.e., 1.25 M and 0.88 vol % of EtOH in THF, was tested with 200, 600, and 1200 ppm of H_2_O for both salts (i.e., LiBF_4_ and LiFOB). The results, in **Table S5**, showed a slight variability between those values. For both salts, the FE decreased at higher H_2_O content, as expected. Notwithstanding this observed slight decrease in the FE, the range of variability between different H_2_O amounts was among the error of each test condition, suggesting that the effect of H_2_O in the studied range is not drastically affecting the system. Moreover, even at ambient pressure, i.e., in N_2_ mass transport limitation conditions, the values obtained with LiFOB were slightly higher than the FE obtained using LiBF_4_, in‐line with the tests performed in the autoclave. Finally, differences in the SEI layer morphology were observed when adding 600 ppm of H_2_O to the LiFOB electrolyte (**Figure S9.e** and **S10.e**), confirming the non‐negligible impact of this parameter.

Based on these findings, for the subsequent experiments, the H_2_O content in the electrolyte was quantified before each test using Karl Fischer titration and adjusted to 600 ppm. In that way, even though the FE will be unavoidably lower than the maximum achievable in the same setup with lower H_2_O content, all unaccounted variations due to H_2_O presence remained as constant as possible across experiments, ensuring precise statistical calculations. This fairly high H_2_O content supported by the batch system is also interesting as it opens up possibilities for a more feasible scale‐up.

#### Fixed‐Parameter Setting: Evaluating the Electrochemical Protocol

The electrochemical protocol, particularly the current density, is a key parameter in determining the selectivity of interfacial reactions, as it affects the lithium electro‐deposited morphology, the SEI layer nature, and the electrode polarization, with a resulting different charge distribution in the double layer.[^12^, ^44^, ^71^, ^72^] The cycling protocol parameters were carefully selected to avoid uncontrolled variations between tests and ensure the statistical relevance to the study.

Particularly, a current pulse was applied before the LSV, as this technique was intrinsically dependent on the internal resistance, manually compensated. With this pulse, all the species could be reduced from the beginning of the test, leading to an electrode/electrolyte configuration similar to the one present during the production step, and more comparable between different tests. An increase in the FE was obtained with this protocol in comparison with performing LSV at first, or a 5 min chronoamperometry (at −3 V *vs* LFP) as first step (**Table S6**).

The total charge passed was also controlled in all tests and set to 20 C, as this value ensured sufficient NH_3_ production for accurate and precise IC detection. Higher charges would extend the test duration without necessarily increasing the total NH_3_ accumulated in the electrolyte. Considering that the experiments were performed in a single‐compartment glass batch cell with a platinum mesh anode, the produced NH_3_ could be oxidized at the anode during the current imposition step.^[12]^ As a matter of fact, a test using the same protocol with LiBF_4_ 1 M and EtOH 1 vol % in THF, but increasing the total passed charge to 30 C, resulted in a lower FE (16 % instead of 22 %). Thus, 20 C was selected as an optimal compromise for this setup.

All the other variables which may influence the process, e.g. the resting time before and after the test, in which spontaneous reactions of the electrolyte components and the electrode interface may start, were maintained constant throughout the study.

#### Study of the Influence of Input Factors on Li‐NRR FE Using Doehlert Design and Response Surface Methodology Analysis

The two‐factor Doehlert design matrix and the respective obtained response variable (i.e., FE) for the two studied salts are summarized in **Table S2** in the Experimental Section.

To validate the statistical consistency of the chosen setup and methodology, the study was previously performed with LiBF_4_, for which a previous study in the same setup reported a maximized FE with an electrolyte composition of LiBF_4_ 2 M with EtOH 1 vol % in THF.^[22]^ Then, the same design was performed with the newly proposed salt (i.e., LiFOB) and the results were finally compared for the two salts.

From data analysis by multiple regression, it is possible to calculate the coefficients of the polynomial model (Eq. S3). The two estimated model parameters for the FE and the two studied salts were:
(1)

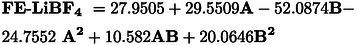



(2)

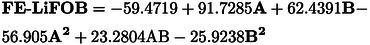




**Figure 5:**
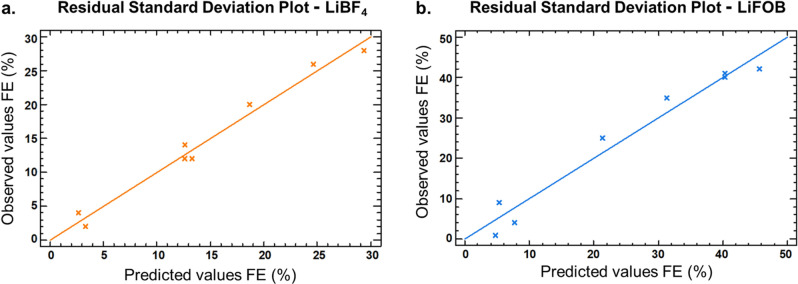
Residual standard deviation plot for the two models obtained with the Doehlert design, with (**a**) LiBF_4_ and (**b**) LiFOB as salt in the electrolyte.

The assessment of the quality, accuracy, and validity of the models was evaluated using the analysis of the variance (ANOVA). The regression models were significant (p<0.05) for both models, the coefficient of determination (R^2^) and the absolute error values were of 0.978 and 1.18 for the LiBF_4_ model, respectively, and 0.965 and 2.6 for the LiFOB one, indicating high precision and repeatability of the statistical models. Moreover, from the residual standard deviation plot (Figure 5), which describes the difference in standard deviations of observed values vs. predicted, it can be said that for both models the predicted values are well matching with the experimental values of FE, as the points in the graph are close to the straight line.

**Figure 6:**
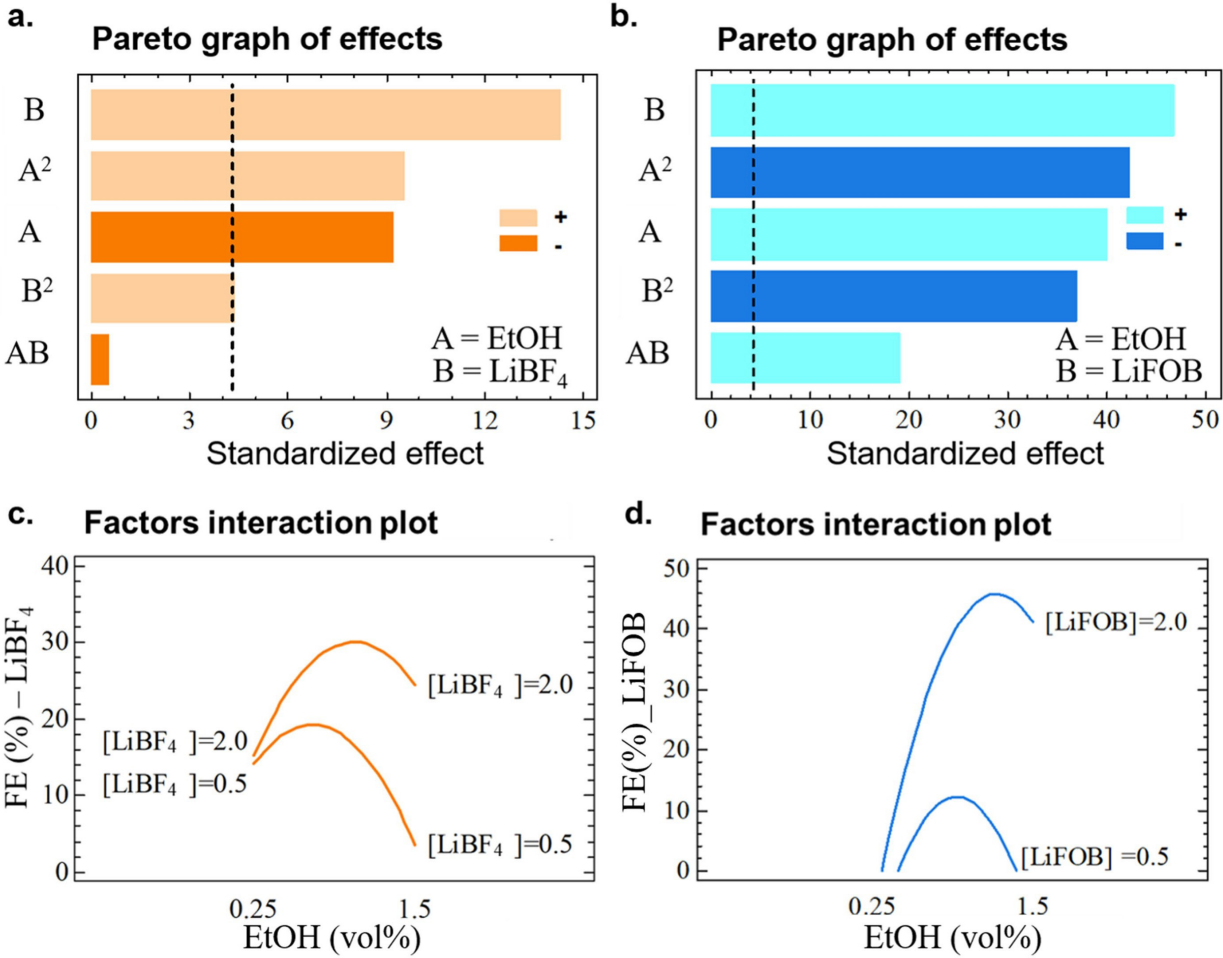
**a,b**. Pareto graph of effects and **c,d** factors interaction plot for the two models obtained with the Doehlert design, with LiBF_4_ (**a** and **c**) and LiFOB (**b** and **d**) as salt in the electrolyte.

The linear and second‐order effects of the selected input factors within the model are represented on the Pareto graph of effects (Figure 6). A Pareto graph displays a frequency histogram with the length of each bar proportional to each estimated standardized effect. The vertical line in the Pareto graph indicates whether each effect is statistically significant within the surface model with p<0.05. Moreover, the graph shows if the effect is positive or negative, which for the linear term means that the FE increases or decreases, respectively, while in the case of quadratic terms it indicates the presence of a minimum (when it is positive) or maximum (when it is negative) in the surface response.

In this study, the salt molarity (factor B) emerged as the most influential factor in determining the system performance, and this was observed both for LiBF_4_ and LiFOB (Figure 6.a,b). For LiBF_4_, the positive quadratic effect B^2^ suggests the presence of a minimum, coherently with the dual effect of molarity explained. However, in the case of LiFOB, its quadratic term (B^2^) is negative, indicating a different surface of response for this salt, with a maximum in the selected range. The presence of a maximum in the LiFOB concentration could be related to the presence of double bonds in the LiFOB molecule (i.e., two C=O groups). As previously hypothesized, the double bonds could lead to a continuous electrolyte degradation, similar to what was observed with LiBOB salt in a flow cell.[^19^, ^22^, ^60^]

As the current density was the same for all the experiments, the amount of plated lithium should be barely affected. As previously detailed, the molarity of the salt is connected with electrolyte conductivity, the polarization of this interface, and thus the double layer and the ion solvation shell.^[72]^ Consequently, it was expected to affect the availability of the different electrolyte species at the cathodic interface, resulting in a different SEI layer composition.[^13^, ^18^, ^19^, ^24^, ^73^] Moreover, the salt nature was confirmed to influence the system even strongly. Lithium plating was observed, from LSV, at a different potential (**Figure S13**). The ionic conductivity was higher for the electrolyte with LiFOB in comparison with LiBF_4_ (**Figure S14**).

Regarding the second studied factor, ethanol (EtOH, A), its influence on the Faradaic efficiency (FE) was less significant compared to the salt molarity. However, its effect varied depending on the salt used. For LiBF_4_, EtOH showed a negative influence, as indicated by the negative sign in the Pareto graph, whereas, for LiFOB, it exhibited a positive effect. The effect of its quadratic terms (A^2^) is negative for both salts, indicating the presence of a maximum (Figure 6.a). However, the combination of EtOH with both salts (AB) has a positive effect. These findings are coherent with the controversial effect of EtOH reported for the Li‐NRR system.[^19^, ^22^]

The fact that the EtOH effect is more influential for the LiFOB electrolyte could be explained considering (i) the competition in the SEI layer formation with this less stable anion and (ii) that a SEI layer with a higher content of organic species could be less compact, thus easily physically destabilized by bubbles evolution on the cathode surface.

This supposition is supported by the factor interaction plot (Figure 6 **.d**), from which small changes in the electrolyte composition drastically affect the FE. A sharp increase in FE is obtained when the amount of both EtOH and LiFOB are high. On the other hand, at low EtOH amount, the predicted FE is very low for the whole range of LiFOB concentration (**Figure S12.b**).

The correlation of a positive effect of EtOH whenever supported by the simultaneous increase of LiBF_4_ was corroborated by the shifting of the maximum observed in the plot of the input factor interaction graph (Figure 6 **.c**). Therefore, for the curve corresponding to predicted point with LiBF_4_ 2 M, the max FE obtainable was expected at a higher EtOH vol% in comparison with the system with LiBF_4_ 0.5 M. Moreover, at 0.25 vol %, the highest FE predicted is the same for both 0.5 and 2 M concentration of LiBF_4_. Fixing the EtOH amount at its extreme value, an inversion is observed, i.e., when the LiBF_4_ concentration is low, the obtained FE is higher for the lowest amount of EtOH, while a higher amount of EtOH combined with LiBF_4_ 0.5 M is detrimental; however, when moving towards higher amount of salt, the highest FE predicted corresponds to 1.5 vol % (**Figure S12.a**).

Summarizing, the role of the EtOH appeared to be more essential when employing LiFOB instead of LiBF_4_, suggesting the need to carefully tailor the competitive degradation of these species and the consequent SEI layer formation. The SEI layer characterization reported in the previous Section corroborated this hypothesis, showing different species when various EtOH amounts were used, both on the surface of the layer and in the depth of the deposit. In particular, in the air‐free XPS (Figure 2) of the sample with the highest EtOH amount tested in the electrolyte (i.e., 1.5 vol %), the relative atomic % of B and O was higher, as well as the intensity of the C 1s peak related to C=O groups, suggesting a SEI layer composition predominantly related to LiFOB decomposition.

These results suggested that, for this newly proposed salt, the identification of the narrow area in which the electrolyte composition enables a stable and bifunctional SEI layer is even more critical. The application of the design of experiments methodology proved to be an effective approach to find this optimal balance, enabling careful optimization of the electrolyte composition to support the formation of a SEI layer well‐suited for the batch system, toward a stable and efficient process.

**Figure 7:**
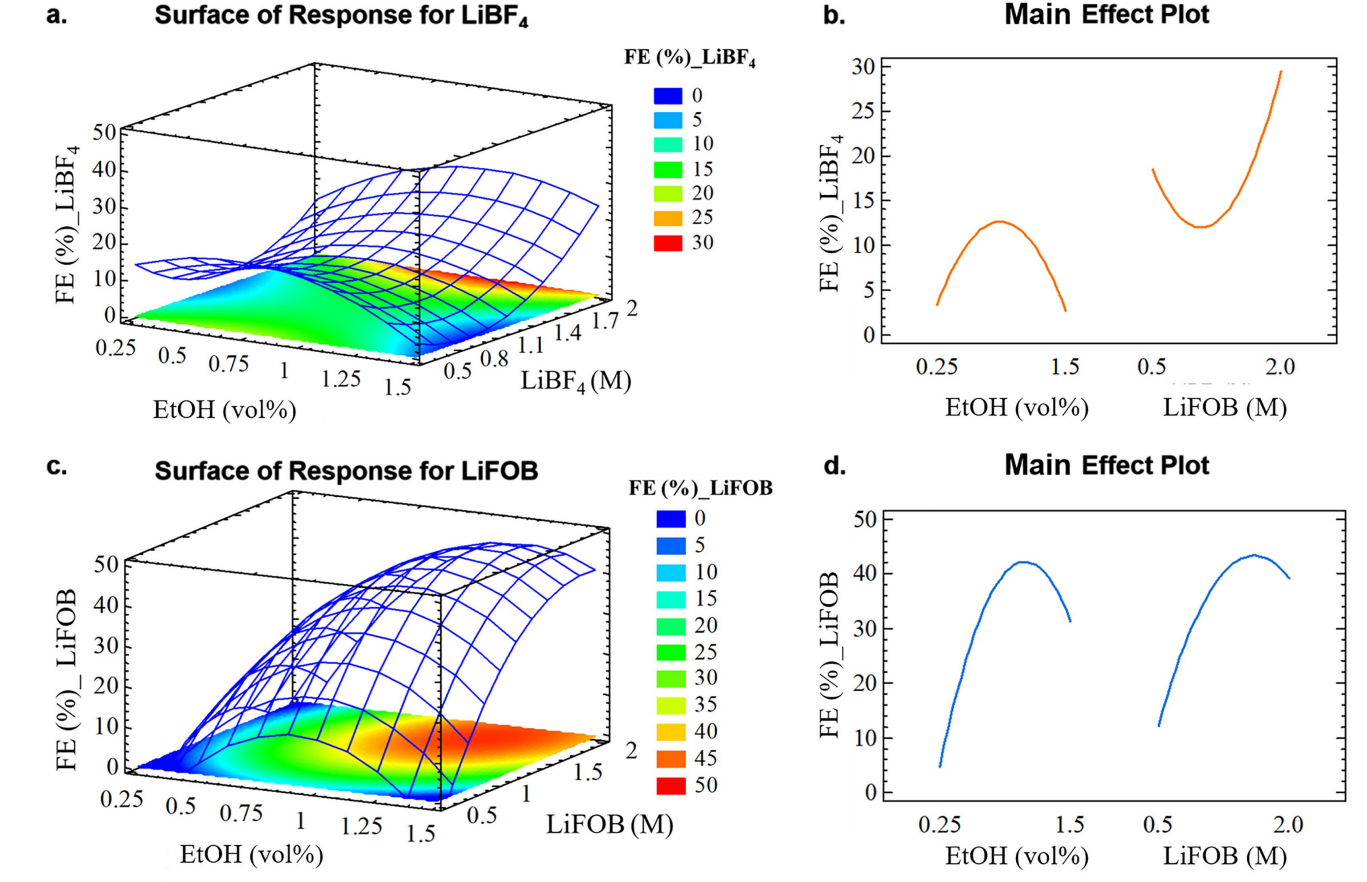
**a., c**. Surface of the response and **b., d**. Main (or principal) effect plot for the two models obtained with the Doehlert design, with LiBF_4_ (**a., b**.) and LiFOB (**c., d**.) as salt in the electrolyte.

The plot of the surface of the response, as well as the main effect plot (Figure 7), were obtained using the quadratic equation for both models, i.e., with LiBF_4_ and LiFOB. Finally, the optimum was calculated fixing the target of a FE of 100 %; the salt concentration and EtOH vol% at this point for both models are reported in Table 1.

**Table 1:** Optimal electrolyte composition in terms of EtOH vol% and Li salt molarity, and respective FE values calculated for the two salts studied (i.e., LiBF_4_ and LiFOB).

	LiBF_4_	LiFOB
EtOH vol% optimal	1.02	1.16
Li salt [M] optimal	2	1.7
FE % optimal point	30	47

The surface and main effect plots for LiBF_4_ (Figure 7 **.a** and Figure 7 **.c**, respectively) revealed divergent trends for the two studied factors, as already discussed, showing a maximum for the EtOH concentration and a minimum for the salt molarity. The FE increased by raising the EtOH concentration up to reaching a maximum at 1.02 vol %, with a subsequent decrease, as previously reported.[^20^, ^22^] Regarding the LiBF_4_ concentration, the previously reported increase in FE with molarity is confirmed.^[22]^ However, a minimum was observed at ca. 0.8 M, suggesting insufficient electrolyte conductivity and ion solvation at this specific concentration, that results in a low FE.^[74]^ On the contrary, at even lower molarity (i.e., LiBF_4_ 0.5 M), these negative effects might be slightly mitigated due to a lower dynamic viscosity of the medium.[^6^, ^56^]

Regarding the LiFOB model (Figure 4 **.b**, **4.d**), the variation of the FE with the two input factors presents a maximum in both cases, as already stated above. In the case of the salt molarity, the maximum FE is obtained at 1.7 M concentration, coherently with the discussed lower stability of the anion and resulting in a general advantage regarding aspects such as the process cost and the possible solubility limits in different solvents. Regarding EtOH, the optimal amount resulted in 1.16 vol %, slightly higher than the amount obtained for LiBF_4_.

Those findings confirmed the complexity of the system, unrevealing hardly predictable relations between the factors and their strong influence on the performances and justifying the application of a statistical method to model the system response also in not tested electrolyte compositions.

The agreement with the literature of the optimal point for the LiBF_4_ electrolyte composition for the same batch setup^[22]^ allowed to validate both the experimental procedure and the statistical methodology employed in the present study. The FE obtained with the model at the optimal point was 30 %, with an error of the model of ±2.6 %, lower than the FEs reported in literature for similar batch setup,[^22^, ^58^] but it should be stressed that the H_2_O content in the system studied was as high as 600 ppm and H_2_O was expected to significantly impact process durability and selectivity,^[17]^ as explained in the previous Section. Moreover, the cathode surface in this work was a flat surface, and a different morphology as a porous cathode or a GDE could affect the electrolyte environment near the cathode and the double‐layer characteristics due to both electrical and steric factors.[^66^, ^75^, ^76^, ^77^, ^78^, ^79^]

Finally, in the case of LiFOB, the FE at the predicted optimum was 47 % ±2.6 %, higher than the one previously predicted for the well‐known LiBF_4_.

In conclusion, with LiFOB the results of the design of experiments analyzed with the response surface methodology showed the possibility of identifying the right compromise between SEI layer stability and permeability by optimizing the electrolyte formulation for the batch system, and with a limited number of experiments. Indeed, in this work only 7 experiments plus two replicates of the central point were enough to predict the behavior of the system in the selected range of the input variables, while in previous studies more experiments were required. Moreover, in such cases the evaluation of possible correlations between factors was not possible. The SEI layer composition was confirmed as crucial for FE optimization, and the utilization of a bifunctional salt as LiFOB, (i.e., giving both LiF and organic components in the SEI layer) showed, when applied in the correct relative amount, an interesting increase in the FE with respect to LiBF_4_ in the batch system, even with a considerable H_2_O impurities content.

## Conclusion

In this work, the Doehlert design methodology has been validated as a powerful tool to fast and effectively optimize the electrolyte formulation for Li‐NRR, and to predict the hidden correlations between the variables affecting the process performance. For that purpose, the widely employed LiBF_4_ was first tested in a well‐known batch setup, an autoclaved single‐compartment glass cell at 20 bar of N_2_, confirming the applicability of design of experiments with response surface methodology as statistical tools to investigate this complex system.

Interestingly, the lithium salt concentration emerged as the most impactful factor in the system, supporting the importance of electrolyte environment at the interface and consequent SEI layer formation. Moreover, the results from the statistical analysis confirmed a sharp variation in the FE with changes in EtOH vol% and the anion chemistry, coherently with the different SEI layer composition observed with chemical‐physical characterizations. The pivotal role of the SEI layer was confirmed, as well as the necessity to control all the cell parameters when designing its formation.

Moreover, the benefits of employing LiFOB as salt in the electrolyte were assessed for the batch setup, reaching a FE higher than the one obtained with LiBF_4_. These improvements are ascribed to the tailoring of a different SEI layer with LiFOB, characterized by a sufficient N_2_ permeability for batch systems, but without compromising the threshold of the necessary mechanical stability of this layer to avoid the continuous electrolyte degradation. Indeed, LiFOB decomposition was expected to form both fluorinated species, desired to stabilize the layer, and organic and oxygenated species, enhancing the affinity to N_2_ of the SEI layer, to achieve the proper reactants ratio on the cathode towards NH_3_ production. Thanks to the response surface methodology analysis, it was possible to specify the optimal electrolyte composition for the studied setup, which was predicted to lead to a FE of 47±2.6 % with LiFOB. The FE obtained was quite remarkable, also considering that, with the aim of better control the H_2_O effect on the system, the amount of this fixed parameter was not negligible, i.e., 600 ppm.

In conclusion, LiFOB is presented as a promising substitute for LiBF_4_ and Doehlert design as an adequate statistical tool to investigate and optimize the electrolyte. As future outlook, this tool could be further implemented to study the effect on the system of additional (up to five) parameters. Interesting parameters that could be deepened to achieve a better understanding of the system are the H_2_O content and the applied electrochemical protocol, which were maintained constant in this study, but resulted to strongly affect the system from preliminary tests. The combination of a GDE at the cathode with a bifunctional salt should also be evaluated, as the different N_2_ gas paths could drastically change the characteristics needed to tailor and optimize the SEI layer. Notwithstanding the complexity of the Li‐NRR system, this multivariate method showed a great potential for further enhancements of performances of NH_3_ electrosynthesis process.

## Supporting Information

The authors have cited additional references within the Supporting Information.

## Conflict of Interests

The authors declare no conflict of interest.

1

## Supporting information

As a service to our authors and readers, this journal provides supporting information supplied by the authors. Such materials are peer reviewed and may be re‐organized for online delivery, but are not copy‐edited or typeset. Technical support issues arising from supporting information (other than missing files) should be addressed to the authors.

Supporting Information

## Data Availability

The data that support the findings of this study are available from the corresponding author upon reasonable request.
