# Supplementary material for: Multivariate Approaches Boosting Lithium‐Mediated Ammonia Electrosynthesis in Different Electrolytes
Source: Angew Chem Int Ed Engl. 2025 Jan 28;64(8):e202416027. doi: 10.1002/anie.202416027 (PMC11833281; doi:10.1002/anie.202416027)
Supplement: Supplementary file 1 — Supporting Information [file ANIE-64-e202416027-s001.pdf]

Angewandte Chemie
Eine Zeitschrift der Gesellschaft Deutscher Chemiker

Supporting Information

Multivariate Approaches Boosting Lithium-Mediated Ammonia Electrosynthesis in Different Electrolytes

A. Mangini, J. B. V. Mygind, S. G. Ballesteros, A. Pedico, M. Armandi, I. Chorkendorff, F. Bella**

Supporting Information

Multivariate Approaches Boosting Lithium-Mediated Ammonia Electrosynthesis in Different Electrolytes

Anna Mangini,[1] Jon Bjarke Valbæk Mygind,[2] Sara Garcia Ballesteros,[1] Alessandro Pedico,[1] Marco Armandi,[1] Ib Chorkendorff,[2] Federico Bella[1]**

1] Department of Applied Science and Technology, Politecnico di Torino, Corso Duca degli Abruzzi 24, Torino, 10129 Italy

2] Department of Physics, Technical University of Denmark, Fysikvej, Kongens Lyngby, 2800 Denmark

** Corresponding authors: sara.garcia@polito.it / federico.bella@polito.it*

Experimental Section

Electrochemical Setup for Design of Experiments Tests

Tests were conducted in a borosilicate single-compartment three-electrode glass cell, placed in an electrochemical home-made autoclave as showed in a previous study,[1] based on the design by Wiberg *et al*.[2] The autoclave was placed in a fume hood. Before each experiment, the cell was cleaned with acetone (ACS reagent, Sigma-Aldrich), boiled in ultra-pure water, and dried overnight at 100 °C in air.

A Pt mesh (1.6 cm_{geo}^2, 99.99%, Goodfellow Cambridge Ltd.) connected with a Pt wire was used as the counter electrode, after being annealed by a butane torch in air. A new flat copper foil with the dimensions of 1.6 cm x 1.0 cm (99.999%, Thermo Fisher Scientific Inc.) was used as the cathode for each experiment, cleaned before the test by dipping in HCl 2 M (VWR International Srl) and rinsed with Milli-Q water. A $Li_{0.5}FePO_4$ (LFP) reference electrode was used, as the stability of this material with various gases has been already demonstrated in similar studies with aprotic electrolytes.[63,64] To this aim, a commercial $LiFePO_4$ powder coated on carbon (MTI Corporation) was chemically partially reduced and adhered to a copper wire (99.999%, Goodfellow Cambridge Ltd.), as previously reported.[5]

The electrolyte solution (10 mL) was prepared in an ILAB Ar-filled glovebox (with impurities value <0.5 ppm H_2O, <0.1 ppm O_2). LiFOB or $LiBF_4$ (99.99%, Sigma-Aldrich, dried at 120 °C under vacuum for 48 h) was added in THF (anhydrous, >99.9%, inhibitor-free, Sigma-Aldrich) to obtain solutions with different molarity. Subsequently, different ethanol amounts (Honeywell International Inc., anhydrous and dried with 4 Å molecular sieves) were added. For the experiments in the presence of FEC (99%, acid <200 ppm, anhydrous, Sigma-Aldrich), an amount corresponding to the 5 wt% of the complete electrolyte was added to the solution.

After the cell electrodes were electrochemically connected, the autoclave was filled with Ar gas (99.999%, Air Liquide), followed by the insertion of the electrolyte solution in the cell. At this point, the H_2O content in the electrolyte was verified by Karl Fischer titration (831 KF Coulometer and 728 Stirrer, Metrohm AG)

and Milli-Q water was added up to 600 ppm, or higher value for the specified tests. Afterward, the autoclave was tightly closed and the internal atmosphere of the autoclave was flushed with N_2 (99.9999%, Air Liquide), increasing the pressure in the autoclave up to 10 bar and de-pressurized to 3 bar for 5 times in order to eliminate any remaining air contaminants; the N_2 gas used was also cleaned by commercial purifiers (NuPure, pptV cleaning of all labile N containing compounds). Finally, the autoclave was filled up to 20 bar and the setup was ready for the experiments.

As a further blank experiment, the autoclave was flushed and filled with Ar to perform the test with the same protocol without N_2 and to verify that no one of the elements in the system was bringing impurities, leading to unwanted NH_3 formation. The test was repeated for each electrolyte at the medium point of the design of experiments ranges, with the same electrochemical protocol, and any NH_3 was measured in the electrolyte after the tests.

Electrochemical Setup for SEI Layer Characterization

An electrochemical single-compartment three-electrode borosilicate glass cell was assembled with the same electrodes and electrolytes preparation procedures previously described, but in this case the cell was assembled and tested in the argon glovebox bubbling N_2 in the electrolyte (5 mL min^{-1}) at ambient pressure during the test, and without adding H_2O in the system. After each test, the cathode was removed from the glass cell in the glovebox and further analyzed with the different procedures as follows.

Electrochemical Measurements

An Sp-200 Biologic potentiostat was used. The electrolyte was saturated for 20 min in N_2, measuring the electrodes potential with the open circuit potential technique. Then, the resistance, required for *iR* correction in the LSV, was determined by electrochemical impedance spectroscopy. After a current pulse at 50 mA cm^{-2}, a LSV at 20 mV s^{-1} was applied to arrive gradually at the onset of lithium deposition (**Figure S13**) before chronopotentiometry (CP), and also to observe eventual addition of reduction peaks and to monitor the system behavior in case of anomalies. Then, NH_3 production was performed with the potential cycling protocol, consisting of alternating 2 min rest period with 1 min CP at 6 mA cm^{-2} until the total passed charge reached 20 C (**Figure S15**). Finally, the autoclaved was slowly degassed bubbling the outcoming gas in an acid trap (20 mL of HCl 0.2 M), but in which any NH_3 was detected, and the electrolyte was collected for NH_3 quantification with by ionic chromatography (IC).

For the SEI layer investigation, the test, performed in the Ar glovebox, consisted of the same protocol described above, but the cycling potential procedure was repeated only 10 times and then the 20 C current was reached with a continuous current application (5 mA cm^{-2}) to avoid the loss of deposited lithium with relative SEI layer after the current imposition.

Ammonia Quantification and Performances Calculation

The produced NH_3 in the electrolyte samples collected after the test was analyzed by means of IC (Metrohm AG), with a method previously employed and validated.[6] The possible volatilized NH_3 was recovered by bubbling the slowing degassing outlet of the autoclave in an acid trap (20 mL of HCl 0.2 M) and the solution was analyzed using IC. No NH_3 was detected in any acid trap analyzed.

Analysis with the IC has been also performed on the washed residue of the cathode surface covered by a thin layer of deposited lithium with the SEI layer, the results showed a negligible amount of NH_3 in the solid phase after the dissolution of these species in acidic H_2O and thus it is not cumulated with the electrolyte NH_3 amount for the FE calculation.

All the FE values were calculated by the equation:

$$FE = 3\ F\ C_{NH_3}\ V\ Q^{-1} \qquad \text{(Eq. S1)}$$

where 3 is the number of electrons transferred for each mole of NH_3, F is the Faraday constant, C_{NH_3} is the concentration of produced NH_3, V is the total electrolyte volume, and Q is the total passed charge.

SEI Layer Characterization

For air-free XPS, the glass cell was tested in the argon glovebox, then samples were transferred to the XPS in a properly sealed transport system to avoid the exposition to air, as previously reported.[8] A Theta Probe equipment (Thermo Fisher Scientific Inc.) was used (Al K_α X-rays source, base pressure below 9.0×10^{-10} mbar), with the ion gun in etching mode (at 4 kV and 1 mA, with scanning size of 2×2 mm^2), flood gun in charge neutralization mode. All the spectra were acquired and analyzed by Thermo Avantage (v5.9925) by Thermo Fisher Scientific Inc. The chamber pressure was 2.0×10^{-7} mbar during the Ar sputtering (99.9999%, Air Liquide). For the survey, the step size was 1.0 eV and dwell time of 50 ms at a pass energy of 200 eV, and the spot size of 400 μm was used. For the high-resolution elemental spectra, the step size was 0.1 eV and dwell time of 50 ms at a pass energy of 200 eV; the spot size was of 400 μm. For calibration, the peak of C 1s at 284.8 eV was used for each spectrum. All the background was determined using Shirley mode and fitted using Powell algorithm. The relative atomic % value was calculated from the fitting of air-free XPS spectrum of each element, each one related to the respective photoionization cross-section value.

For the XRD measurements, the deposited layer was scratched from the cathode inside the argon glovebox and loaded into a commercial air-free domed sample holder (Malvern Panalytical Ltd) with an air-tight PEEK dome. XRD patterns were recorded on an Empyrean X-rays diffractometer (Cu K_α radiation, Malvern Panalytical Ltd) operated at 40 kV and 40 mA. Data were collected with a 2D solid state detector (PIXcel) from 10 to 80° with a step size of 0.026° s^{-1}.

For ATR-FTIR measurements, the copper cathode with the lithium deposit covered by SEI layer obtained after the not autoclaved glass cell experiment was separated from the cell in an Ar-filled glovebox, dried (the THF rapidly vaporized at environmental conditions), and stored in a vial under Ar atmosphere until the measurement was performed. The IR spectra were recorded at 2 cm^{-1} resolution on an Equinox-55 spectrometer (Bruker) equipped with a mercury cadmium telluride cryodetector. Different points of the sample

were studied to ensure the detection a of medium composition of the layer. Different spectra were collected for the same point at different times, to observe the modifications on the sample caused by the air exposition, as represented in **Figure S16**.

For the SEM analysis, the sample of the copper cathode with the lithium deposit covered by SEI layer obtained after the not autoclaved glass cell experiment was separated from the cell in an Ar-filled glovebox, washed with a few drops of THF, and attached to the sample holder pins with carbon tape. The assembled pins were transported to the instrument in an air-tight holder. To minimize the exposure time of the samples to the air, a setup made of an aluminum vessel was arranged on the SEM sample holder, on which the pins were rapidly fixed pouring argon gas in the vessel. The assembled setup was inserted inside the SEM chamber (**Figure S17.b**), and the pump were immediately turned on, preserving the samples in vacuum. After the measurement, the samples appeared of the same color as they were observed in the glovebox (**Figure S17.a**). Electron microscopy characterization was carried out with a field-emission scanning electron microscope (FESEM Supra 40, manufactured by Zeiss) equipped with a Si(Li) detector (Oxford Instruments) for energy-dispersive X-rays (EDX) spectroscopy.

Experimental Design: Doehlert Matrix

The impact of factors towards system response was estimated employing a two-factor Doehlert design, which consists of one central point and six points forming a regular hexagon.[9] Salt concentration and EtOH content in the electrolyte were chosen as input factors/variables, while the FE was chosen as the output or response variable. The number of experiments (N) is given by:

$$N = k^2 + k + C_0 \qquad \text{(Eq. S2)}$$

where *k* is the number of factors (input variables) and C_0 is the number of tests performed at the central points.

In the present study, the number of input variables *k* was 2 (X_1 = salt concentration and X_2 = EtOH content) and 3 replicates of the central point were added to estimate the experimental variance and, thus, validate the model. That way, the total number of experiments generated was 9.

As imposed by the Doehlert method, each input variable (X_i) was studied at a different level of detail: the salt concentration was studied at 3 levels, while the EtOH content in 5. The maximum (X_{max}) and minimum (X_{min}) values selected for each variable define the experimental range of each input factor and, thus, the overall experimental domain (**Table S1**).

Table S1. Coded factors and experimental range selected for each input factor used for the Doehlert matrix.

Input factor	Coded factor	Experimental domain	
		X_{min}	X_{max}
EtOH content (vol%)	X_1	0.25	1.5
Salt concentration (M)	X_2	0.5	2

Table S2. Doehlert matrix in coded and experimental values for each experiment and values obtained for the response variable in every experiment.

**Exp nº**	**Coded values**		**Experimental values**		**Response variable**	
	**X1**	**X2**	**EtOH (vol%)**	**Li salt (M)**	**FE% ($LiBF_4$)**	**FE% (LiFOB)**
1	0	0	0.88	1.25	**12**	**40**
2	1.0	0	1.50	1.25	**4**	**35**
3	0.5	0.817	1.19	2.00	**28**	**42**
4	-1.0	0.000	0.25	1.25	**2**	**1**
5	-0.5	-0.817	0.56	0.50	**20**	**9**
6	0.5	-0.817	1.19	0.50	**12**	**4**
7	-0.5	0.817	0.56	2.00	**26**	**25**
8	0.0	0.000	0.88	1.25	**12**	40
9	0.0	0.000	0.88	1.25	**14**	38

This experimental design and response surface methodology were performed by using StatGraphic Centurion 19 software. The experimental data were matched with the second-order polynomial model:

$$Y = b_0 + b_1X_1 + b_2X_2 + b_{11}X_1^2 + b_{22}X_2^2 + b_{12}X_1X_2 \quad \text{(Eq. S3)}$$

where b_i are the estimation of the main effect of the factors (linear effects), b_{ii} considers the second-order effect, and b_{ij} gives information about the interaction between the input factors.

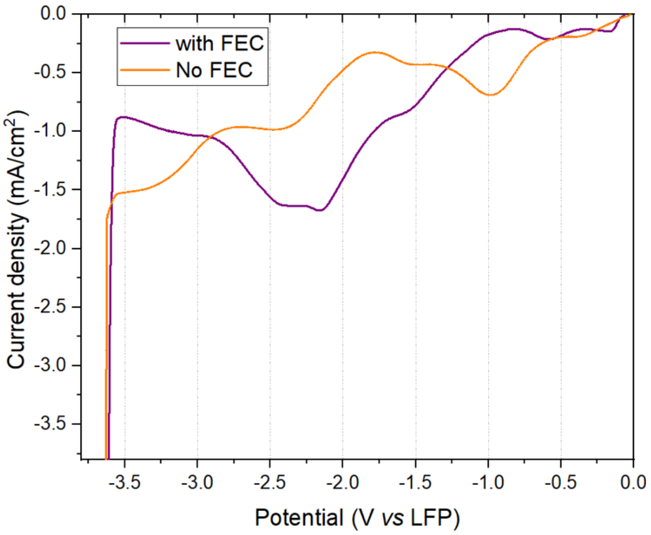

Figure S1. LSV for two electrochemical tests in the autoclaved glass cell with Pt mesh as counter electrode, $Li_{0.5}FePO_4$ as a reference electrode, and copper foil as cathode. The electrolyte composition was $LiBF_4$ 1 M with EtOH 1 vol% and FEC 5 wt% in THF (purple line); the same electrolyte (but without FEC) is shown in orange. The applied scan rate was 20 mV s^{-1} for both tests.

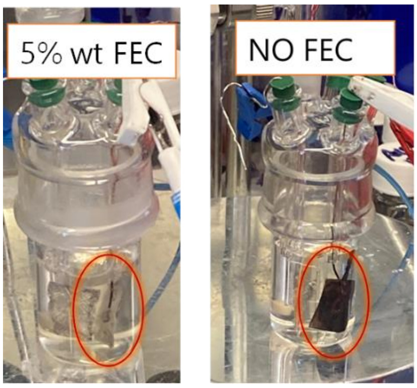

Figure S2. Photos of two electrochemical glass cells after a Li-NRR test performed in the Ar-filled glovebox bubbling N_2 at ambient pressure. The electrolyte composition was $LiBF_4$ 1 M with EtOH 1 vol% (on the right) and with FEC 5 wt% (on the left) in THF. The red circles indicate the deposit obtained after the Li-NRR test: with FEC 5 wt% addition in the electrolyte, the deposit was pale-grey color, while without FEC it was black.

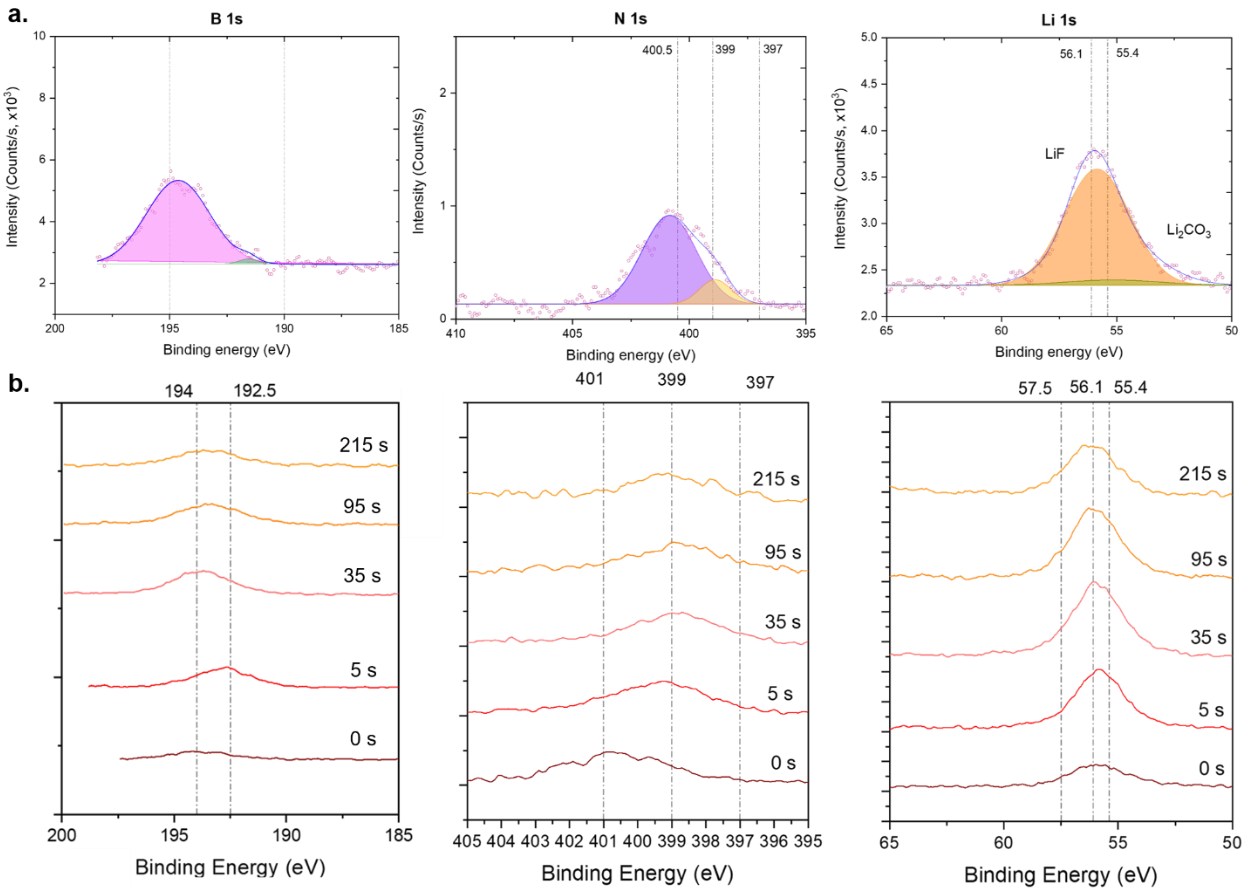

Figure S3. **a.** Air-free XPS elemental spectra of B 1s, N 1s, and Li 1s, with peak fitting and **b.** respective depth profile at different Ar sputtering times. The blue line in the peak fitting (**a**) corresponds to the cumulative peak fit, while the red dot is the raw data. The peak at 284.4 eV of C 1s was used for calibration. All the samples of deposited lithium with SEI layer were obtained on the copper cathode after a Li-NRR test in an ambient pressure batch cell, with LiFOB 1.25 M and EtOH 0.88 vol% in THF as the electrolyte.

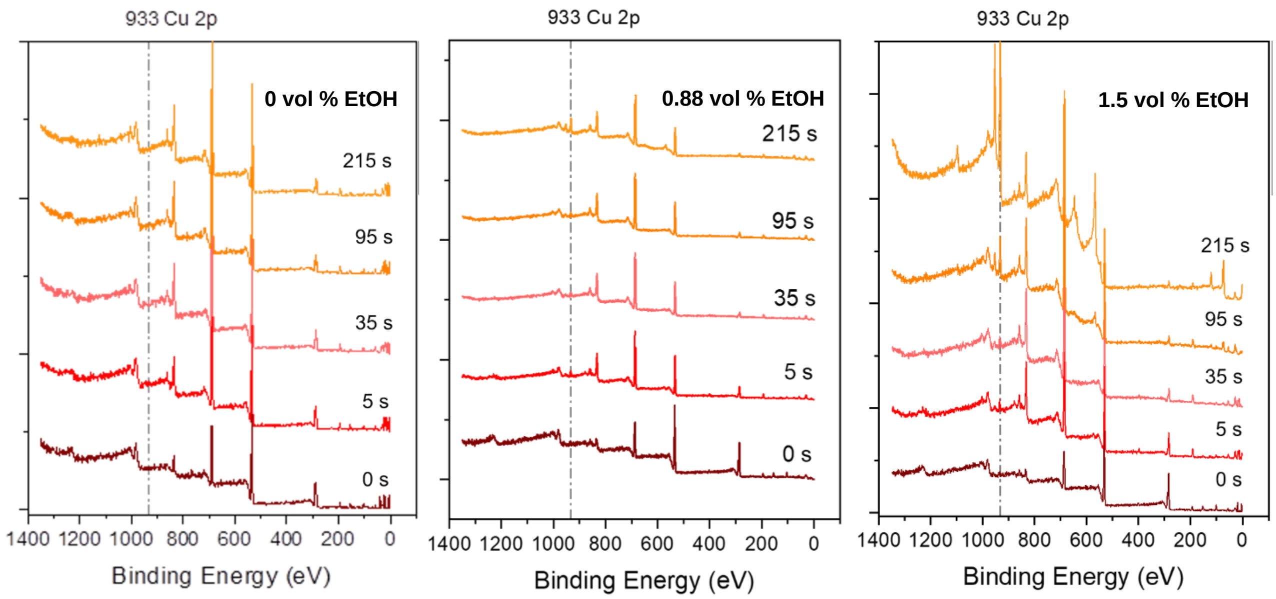

Figure S4. Air-free XPS surveys spectra at different Ar sputtering times of deposit and SEI layer samples obtained after a Li-NRR test in an ambient pressure batch cell, with different EtOH contents in the electrolyte (*i.e.*, from left to right, 0, 0.88, and 1.5 vol%) based on LiFOB 1.25 M in THF. The dashed line highlights the presence of Cu 2p peaks at higher Ar sputtering times in the survey spectra.

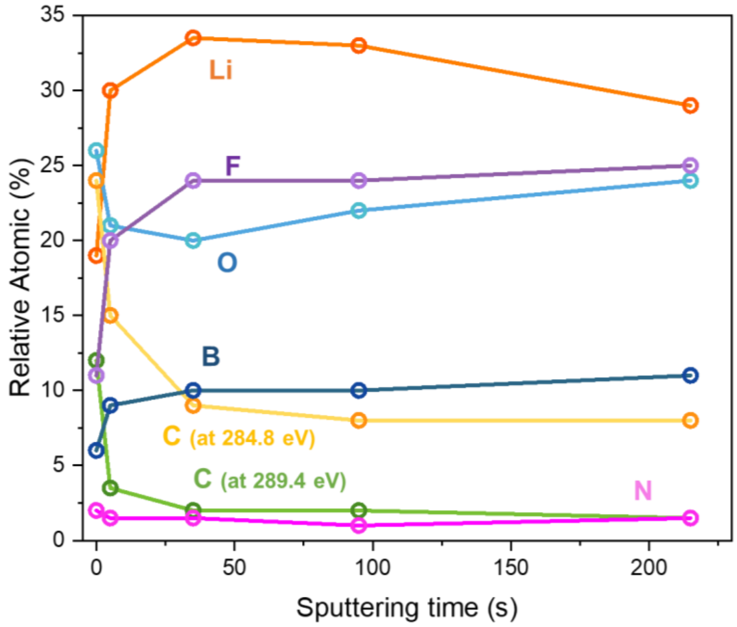

Figure S5. Air-free XPS depth profile comparison for different elements at different Ar sputtering times obtained for a sample of deposited lithium with SEI layer after a Li-NRR test in an ambient pressure batch cell, with LiFOB 1.25 M and EtOH 0.88 vol% in THF as the electrolyte.

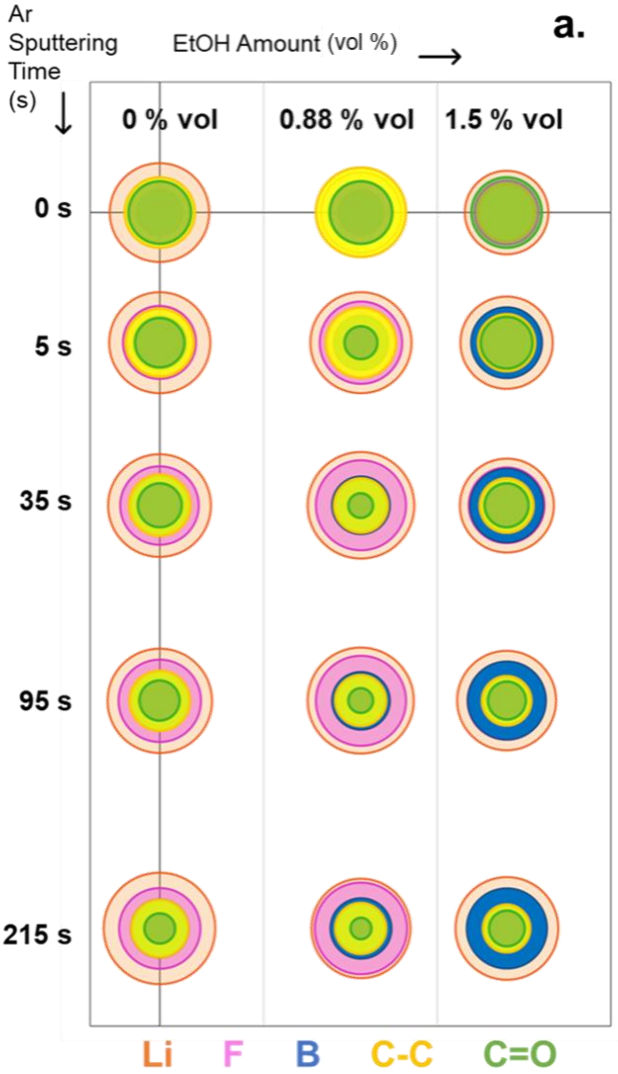

Figure S6. Comprehensive visualization of air-free XPS relative atomic % (corresponding to the circles area) for different elements (related to different colors), compared at different Ar sputtering times (y-axis) for different samples (x-axis: EtOH vol% in the electrolyte used to produce the sample). The samples of deposited lithium with SEI layer were obtained after a Li-NRR test in the ambient pressure batch cell, with LiFOB 1.25 M in THF as the electrolyte. From the left, EtOH amount was of 0, 0.88, and 1.5 vol%. The data presented are resumed in **Table S1**.

Table S3. Relative atomic % at different Ar sputtering times obtained with air-free XPS % for each element for the three different EtOH concentrations tested (0, 0.88, and 1.5 vol %).

NO EToH	Sputtering time:	0	5	35	95	215	0.88 % vol	Sputtering time:	0	5	35	95	215	1.5 % vol	Sputtering time:	0	5	35	95	215
	**F**	10.5	15.8	18.3	20	19.5		**F**	11	20	24	24	25		**F**	12	13.5	17	18	18
	**N**	0.3	0.2	0.2	0	0		**N**	2	1.5	1.5	1	1.5		**N**	0.3	0.2	0	0	0
	**Li**	29	30	31	32	36.5		**Li**	19	30	33.5	33	29		**Li**	20.7	25	26	29	31
	**B**	6.2	8.5	10	10	10		**B**	6	9	10	10	11		**B**	8	15	16	18	19
	**O**	27	24	23	22	21		**O**	26	21	20	22	24		**O**	34	28	26	23	21
	**C (284.8)**	15	14	11.5	11	10		**C (284.8)**	24	15	9	8	8		**C (284.8)**	10	10	9	7.5	7
	**C (291)**	12	7.5	6	5	3		**C (291)**	12	3.5	2	2	1.5		**C (291)**	15	8.3	6	4.5	4

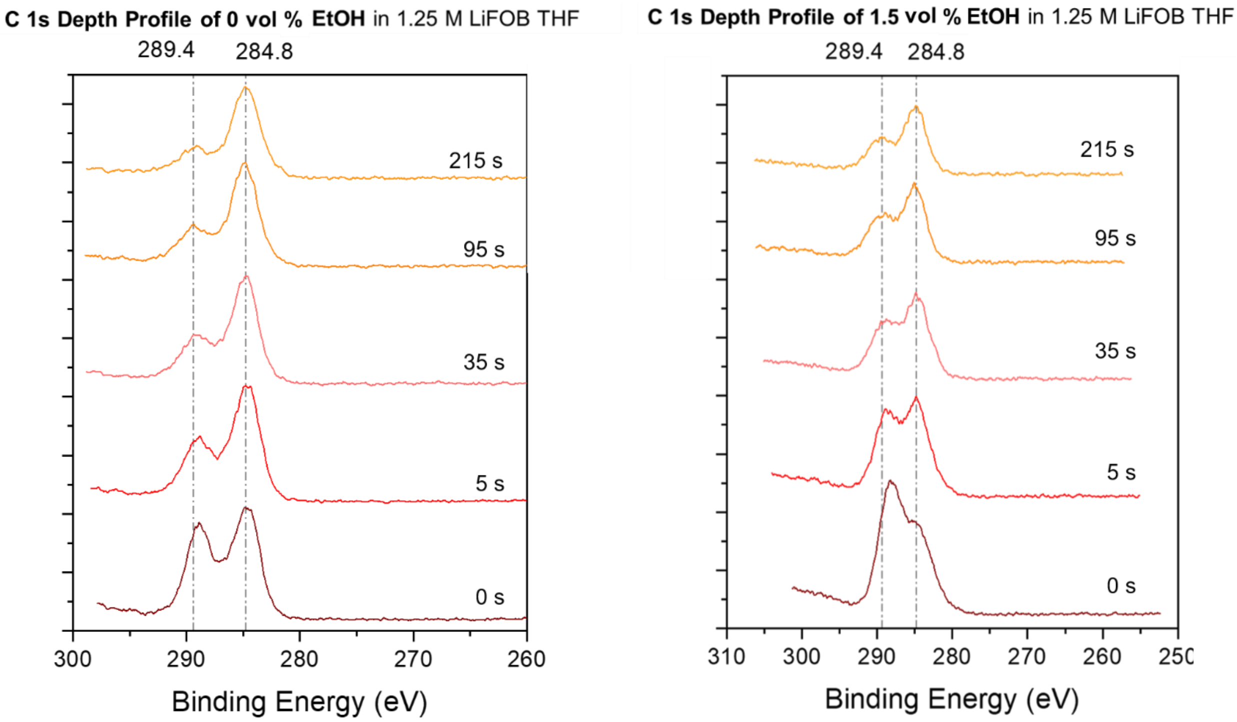

Figure S7. C 1s spectra depth profile at different Ar sputtering times, obtained from a sample of a deposited lithium with SEI layer after a Li-NRR test at ambient pressure in batch cell, with LiFOB 1.25 M and EtOH 0 vol% (left) and 1.5 vol% (right) in THF as electrolyte.

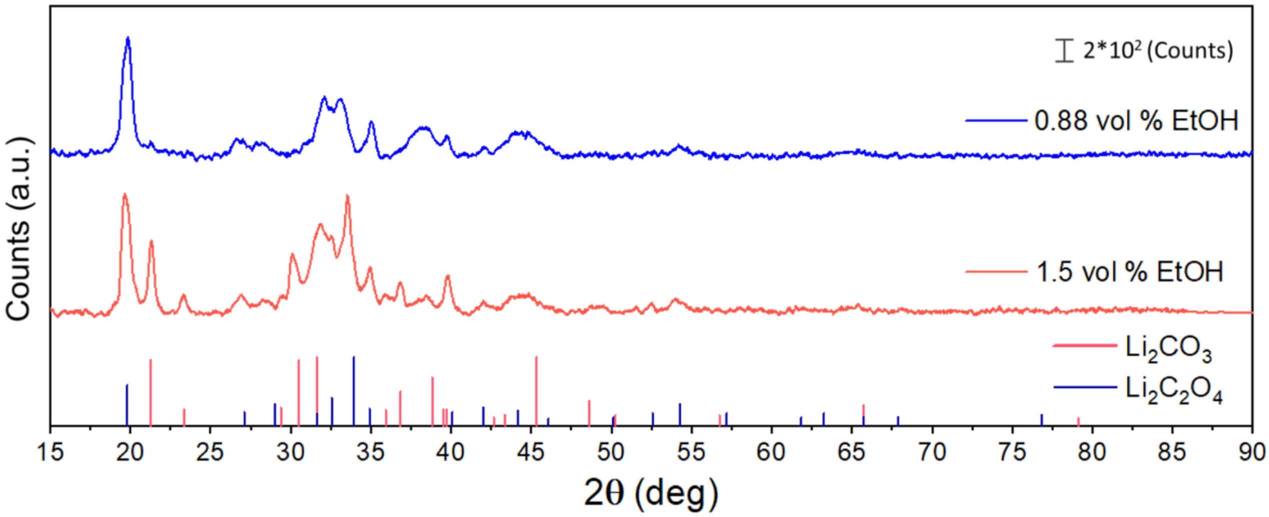

Figure S8. XRD patterns of deposited lithium with SEI layer on the copper cathode obtained after a Li-NRR electrochemical test in the Ar-assembled glass cell and tested bubbling N_2 at ambient pressure, with LiFOB 1.25 M in THF as the electrolyte, with EtOH 0.88 vol% (blue pattern) or 1.5 vol% (pink pattern).

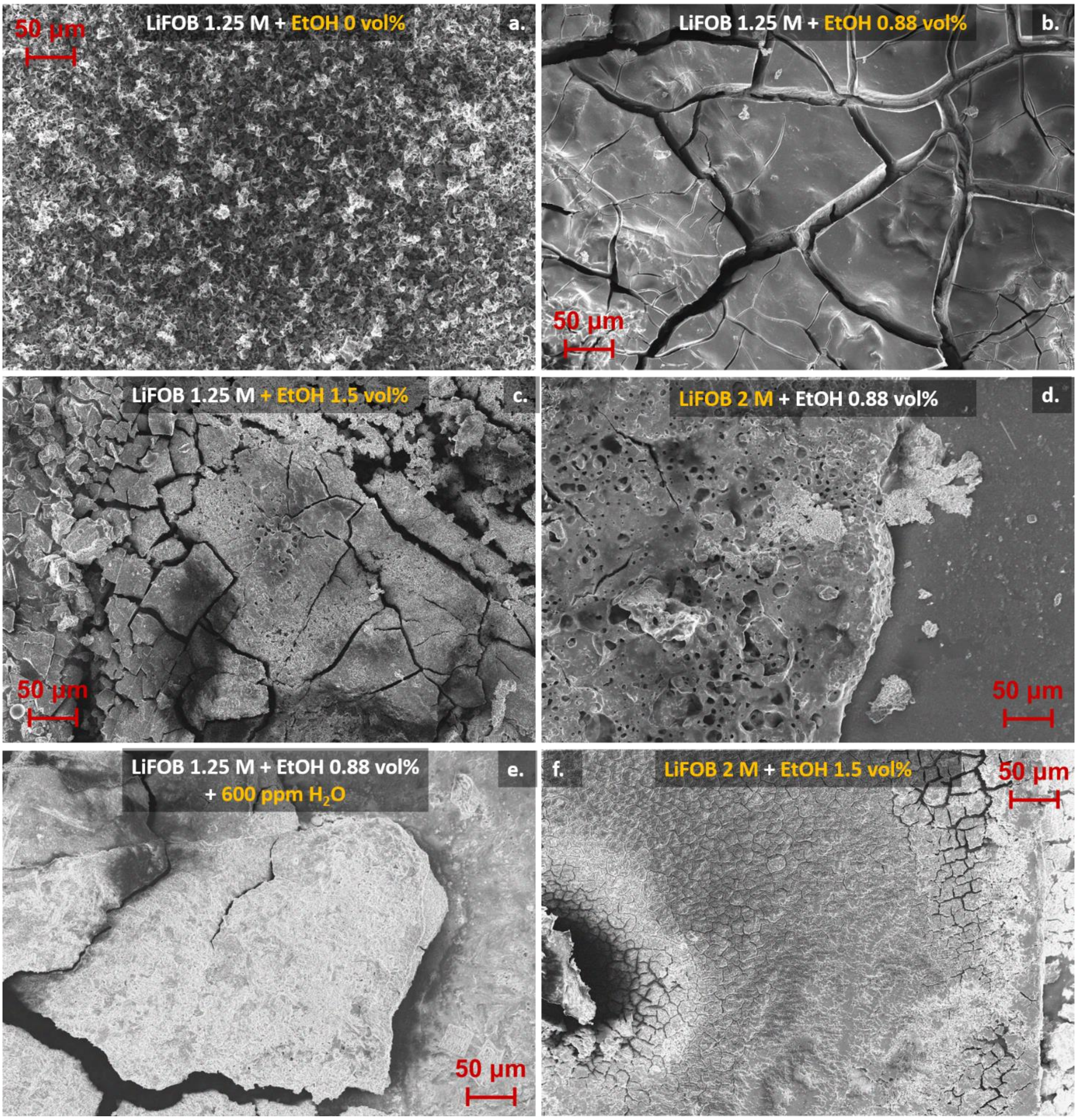

Figure S9. SEM images of the of deposited lithium with SEI layer obtained on the copper cathode after a Li-NRR test at ambient pressure, at a lower magnification with respect of the images in the main text, with an electrolyte composed of: **a.** LiFOB 1.25 M in THF, **b.** LiFOB 1.25 M with EtOH 0.88 vol% in THF, **c.** LiFOB 1.25 M with EtOH 1.5 vol% in THF, **d.** LiFOB 2 M with EtOH 0.88 vol% in THF, **e.** LiFOB 1.25 M with EtOH 0.88 vol% in THF and 600 ppm of H_2O, and **f.** LiFOB 2 M with EtOH 1.5 vol% in THF.

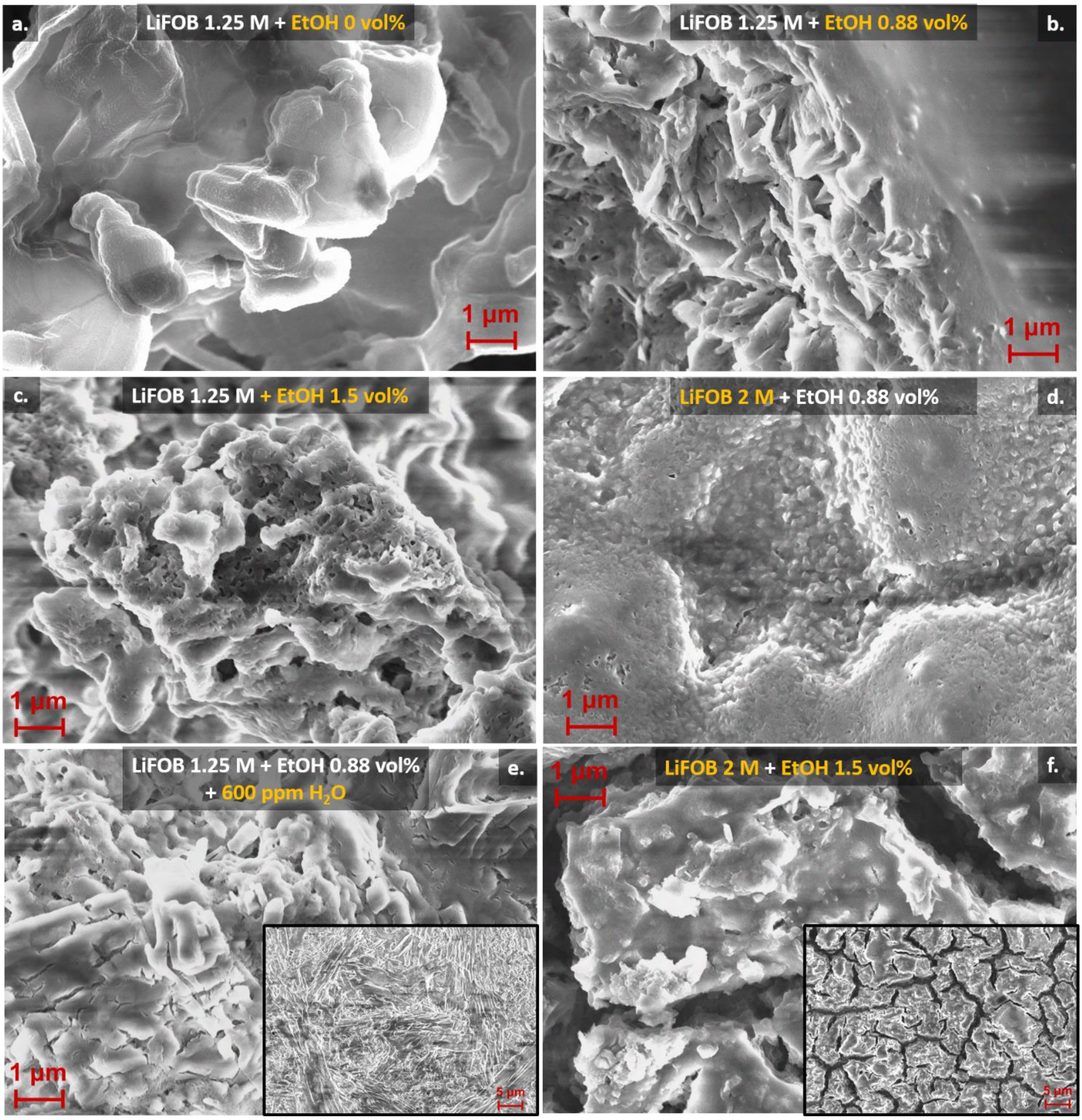

Figure S10. SEM images of the of deposited lithium with SEI layer obtained on the copper cathode after a Li-NRR test at ambient pressure, at a higher magnification with respect of the images in the main text, with an electrolyte composed of: **a.** LiFOB 1.25 M in THF, **b.** LiFOB 1.25 M with EtOH 0.88 vol% in THF, **c.** LiFOB 1.25 M with EtOH 1.5 vol% in THF, **d.** LiFOB 2 M with EtOH 0.88 vol% in THF, **e.** LiFOB 1.25 M with EtOH 0.88 vol% in THF and 600 ppm of H_2O, and **f.** LiFOB 2 M with EtOH 1.5 vol% in THF. Insets in **e.** and **f**. show the images at lower magnification.

Table S4. Reduction potentials for the molecules evaluated in this study, *i.e.*, THF, used as solvent, the salts $LiBF_4$ and LiFOB, and the additives EtOH and FEC. In the second column, the values obtained *vs* SHE, and in the third column, the values experimentally measured *vs* Li^+/Li in carbonate-based electrolyte; in the fourth column, the respective reference. It should be noted that the values of the reduction potential of the species in the specific system of this work might not accurately be represented from the value reported in this table, as they were obtained in a different electrolyte environment, and on different electrode.[10,11] The actual potential could vary of 0.5 V.[10]

	**V *vs* SHE**	**V *vs* Li^+/Li**	**Reference**
**THF**		-1.5	[11]
**$LiBF_4$**		0.5	[12]
**LiFOB**		1.6	[13]
**H_2O**		1.5	[14]
**FEC**		1.4	[15]
**EtOH**	-1.1		[16]

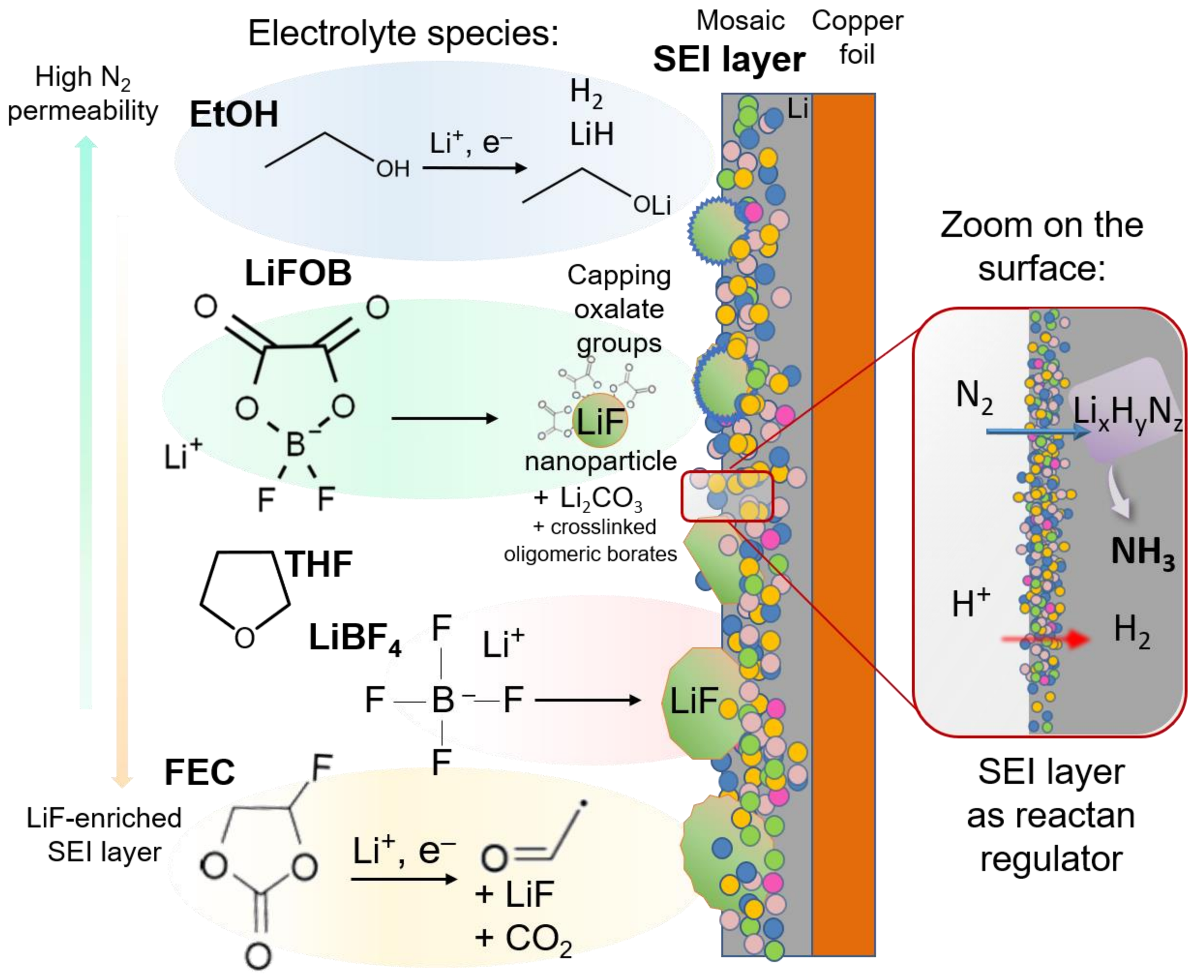

Figure S11. Schematic representation of the possible reaction of each electrolyte component on the cathodic surface, contributing to the SEI layer formation. On the red zoom: graphical representation of the obtained SEI layer as a regulator of the reactant species on the electrode surface, where lithium is plated.

Table S5. FE dependence on H_2O amount: FE obtained from Li-NRR tests with different contents of H_2O (ppm), different repetitions were present. **a.** Tests performed in the autoclaved glass cell. The electrolyte composition was $LiBF_4$ 1 M with EtOH 1 vol% in THF. **b., c.** Test performed in the glass cell at ambient pressure, with $LiBF_4$ (**b.**) or LiFOB (**c.**) 1.25 M with EtOH 0.88 vol% in THF, with 200, 600, or 1200 ppm of H_2O.

**a.** Autoclaved cell<br>**$LIBF_4$ 1 M with EtOH 1 vol% in THF**			
**H_2O content (ppm):**	**200**	**1000**	**2000**
**FE (%):**	22	19 ± 2	0
**b.** Not-autoclaved cell<br>**$LIBF_4$ 1.25 M with EtOH 0.88 vol% in THF**			
**H_2O content (ppm):**	**200**	**600**	**1200**
**FE (%):**	9 ± 3	7 ± 3	6 ± 2
**c.** Not-autoclaved cell<br>**LiFOB 1.25 M with EtOH 0.88 vol% in THF**			
**H_2O content (ppm):**	**200**	**600**	**1200**
**FE (%):**	12 ± 3	8 ± 2	7 ± 2

Table S6. FE obtained in Li-NRR tests with different electrochemical technique applied before the normal protocol, *i.e.*, the one detailed in the experimental section for the autoclaved cell. The different first technique applied corresponds to the one specified for each column. The electrolyte was, for all the test, $LiBF_4$ 1 M with EtOH 1 vol% in THF.

Autoclaved cell<br>**$LIBF_4$ 1 M with EtOH 1 vol% in THF**			
**I step of the protocol technique:**	**Current pulse**<br>50 mA cm^{-2} for 1 s	**LSV**<br>20 mV s^{-1}	**Chronoamperometry**<br>at –3 V *vs* LFP
**FE (%):**	22	12	14

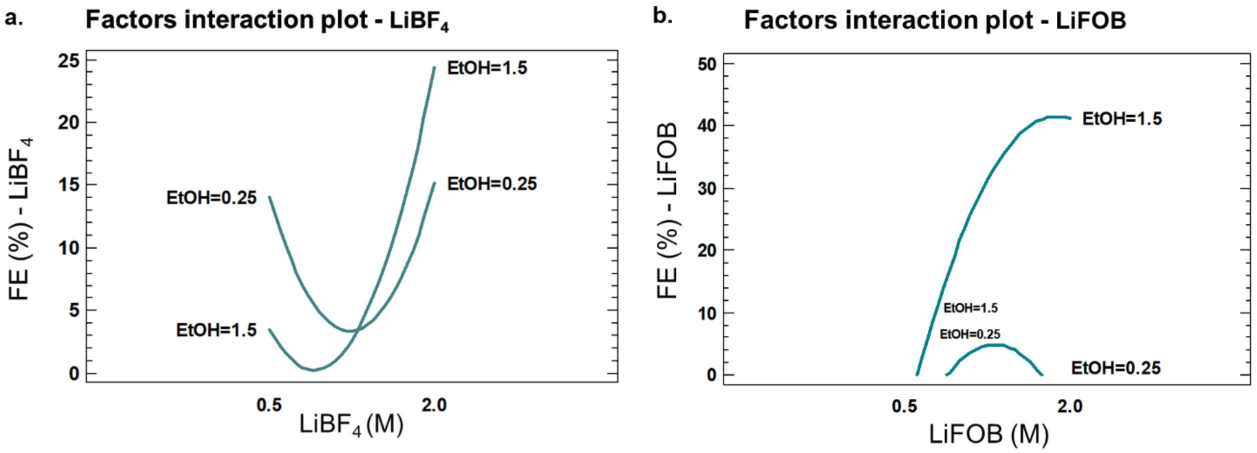

Figure S12. Selected input factor interaction for the two models obtained with the Doehlert design and response surface methodology: $LiBF_4$ (**a**) and LiFOB (**b**) as salt in the electrolyte.

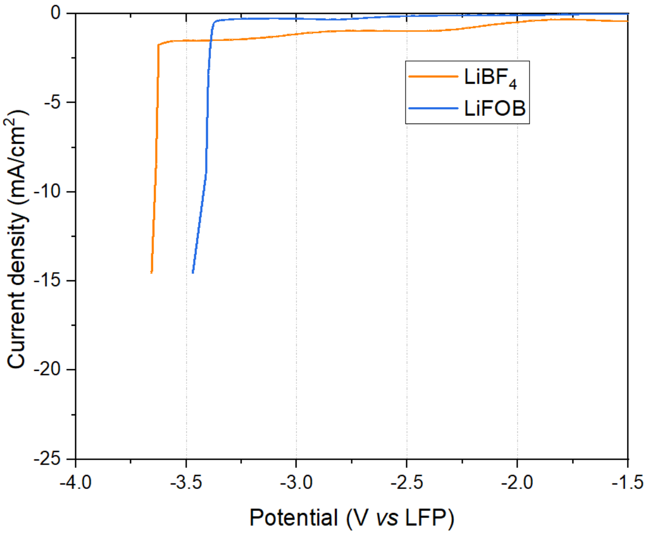

Figure S13. LSV for two electrochemical tests (corresponding to the central point of the Doehlert matrix) performed in the autoclaved glass cell with Pt mesh as counter electrode, LFP as reference electrode, and copper foil as cathode. The electrolyte used was $LiBF_4$ 1.25 M with EtOH 0.88 vol% in THF for the orange line and LiFOB 1.25 M with EtOH 0.88 vol% in THF for the blue one. The applied scan rate was of 20 mV s^{-1} for both tests.

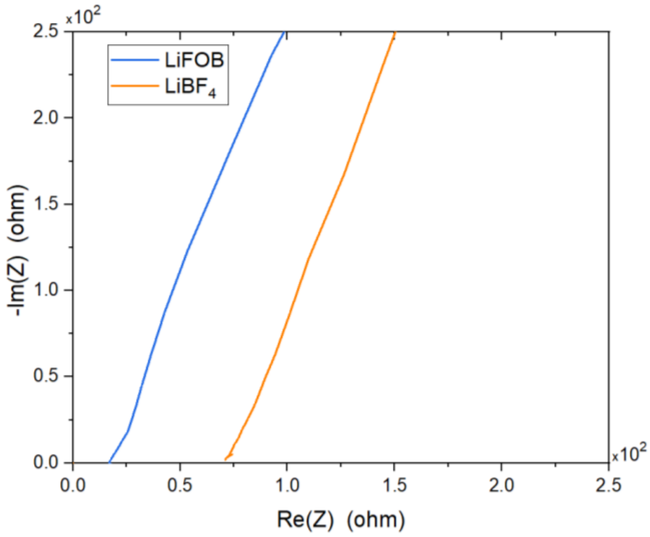

Figure S14. Nyquist plots obtained from the preliminary electrochemical impedance spectroscopy (with perturbation of the potential) of two electrochemical tests (corresponding to the central point of the Doehlert matrix) performed in the autoclaved glass cell with Pt mesh as counter electrode, LFP as reference electrode, and copper foil as a cathode. The electrolyte used was $LiBF_4$ 1.25 M with EtOH 0.88 vol% in THF for the orange line and LiFOB 1.25 M with EtOH 0.88 vol% in THF for the blue one. The scan rate applied was of 20 mV s^{-1} for both tests.

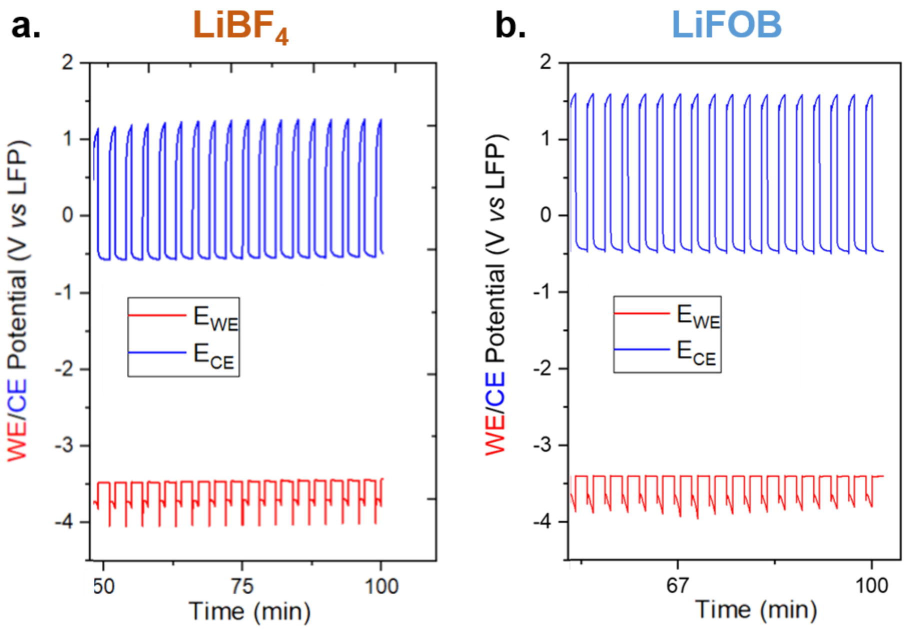

Figure S15. Example of the potential cycling protocol applied in all experiments for the design of experiment tests with different electrolyte compositions. The two electrochemical tests reported correspond to the central point of the Doehlert matrix, *i.e.*, using (**a**.) $LiBF_4$ 1.25 M and (**b**.) LiFOB 1.25 M with EtOH 0.88 vol% in THF as electrolyte in the pressurized batch glass cell at 20 bar. The current imposition (6 mA cm^{-2}) for 1 min was alternated with 2 min of rest, in which the open circuit voltage was registered, specifying the potential of the cathode, or working electrode (WE), and at the anode, or counter electrode (CE), *vs* the reference electrode made of $Li_{0.5}FePO_4$. All potentials are without iR correction.

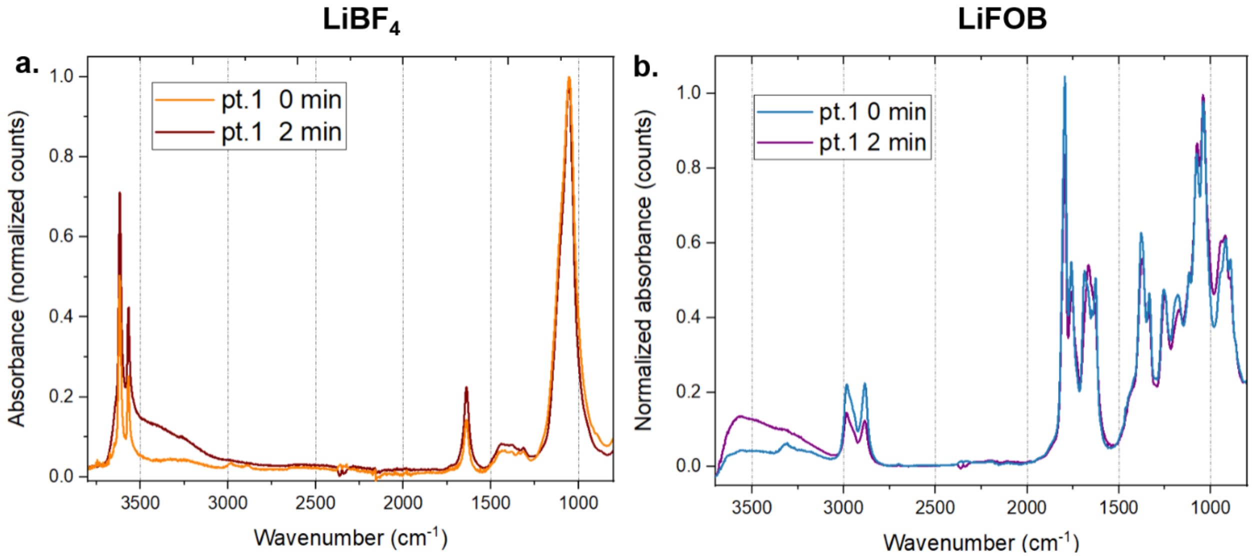

Figure S16. ATR-FTIR spectra of the same point of the same sample of deposited lithium with SEI layer on the copper WE, at different times. The sample was obtained after an electrochemical test conducted in the glass cell assembled in Ar with the flat copper cathode, a Pt mesh as counter electrode, and LFP as reference. The electrolyte composition was (**a**) $LiBF_4$ 1.25 M or (**b**) LiFOB and EtOH 0.88 vol% in THF, *i.e.*, the central point in the design of experiment. After 2 min, it was possible to observe a broad additional band between 3000 cm^{-1} and 3500 cm^{-1}, corresponding to the increase of the water interacting by H-bonding on the sample. The nature of the rest of the peaks did not change after air exposition, suggesting a sufficient stability of the sample. The absorbances were normalized by dividing for the maximum of each curve.

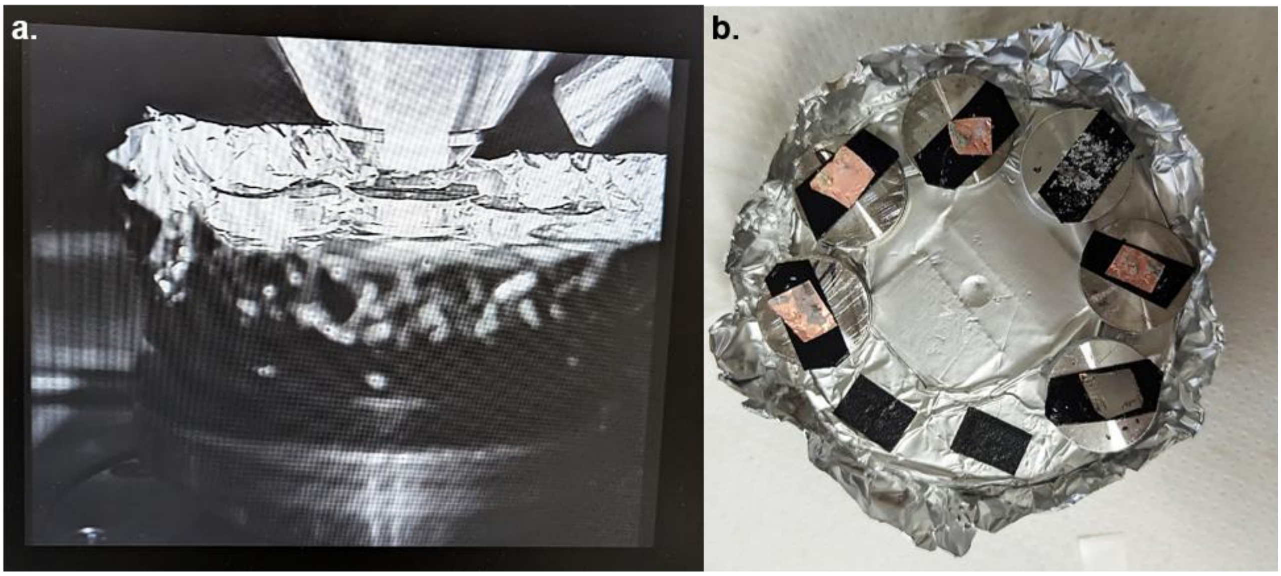

Figure S17: a. Samples inserted inside the SEM chamber (picture taken during measurements). **b.** Samples mounted on the sample holder, after the measurement. It is possible to see the presence of the aluminum vessel, and notice a dark-grey color, suggesting a minimized oxidation of the samples (from right to left, samples in **Figure 3** and **S9** and **S10**, **a.** to **e.**). It should be notice that the third sample (letter **c** in **Figure 3** and **S9** and **S10**, was detached from the copper foil, as it was so fragile that it broke during the pin preparation. After many minutes, the samples were all of white color.

References

[1] K. Li, S. Z. Andersen, M. J. Statt, M. Saccoccio, V. J. Bukas, K. Krempl, R. Sažinas, J. B. Pedersen, V. Shadravan, Y. Zhou, D. Chakraborty, J. Kibsgaard, P. C. K. Vesborg, J. K. Nørskov, I. Chorkendorff, *Science* **2021**, *374*, 1593–1597.

[2] G. K. H. Wiberg, M. J. Fleige, M. Arenz, *Rev. Sci. Instrum.* **2014**, *85*, 085105.

[3] E. J. Mcshane, P. Benedek, V. A. Niemann, S. J. Blair, G. A. Kamat, A. C. Nielander, T. F. Jaramillo, M. Cargnello, *ACS Energy Lett.* **2023**, *8*, 230–235.

[4] R. Tort, O. Westhead, M. Spry, B. J. V. Davies, M. P. Ryan, M. M. Titirici, I. E. L. Stephens, *ACS Energy Lett.* **2023**, *8*, 1003–1009.

[5] J. B. V. Mygind, J. B. Pedersen, K. Li, N. H. Deissler, M. Saccoccio, X. Fu, S. Li, R. Sažinas, S. Z. Andersen, K. Enemark-Rasmussen, P. C. K. Vesborg, J. Doganli-Kibsgaard, I. Chorkendorff, *ChemSusChem* **2023**, *16*, e202301011.

[6] X. Fu, J. B. Pedersen, Y. Zhou, M. Saccoccio, S. Li, R. Sažinas, K. Li, S. Z. Andersen, A. Xu, N. H. Deissler, J. B. V. Mygind, C. Wei, J. Kibsgaard, P. C. K. Vesborg, J. K. Nørskov, I. Chorkendorff, *Science* **2023**, *379*, 707–712.

[7] X. Fu, S. Li, N. H. Deissler, J. B. V. Mygind, J. Kibsgaard, I. Chorkendorff, *ACS Energy Lett.* **2024**, *9*, 3790–3795.

[8] S. Li, Y. Zhou, K. Li, M. Saccoccio, R. Sažinas, S. Z. Andersen, J. B. Pedersen, X. Fu, V. Shadravan, D. Chakraborty, J. Kibsgaard, P. C. K. Vesborg, J. K. Nørskov, I. Chorkendorff, *Joule* **2022**, *6*, 2083–2101.

[9] M. A. Bezerra, R. E. Santelli, E. P. Oliveira, L. S. Villar, L. A. Escaleira, *Talanta* **2008**, *76*, 965–977.

[10] H. Iriawan, A. Herzog, S. Yu, N. Ceribelli, Y. Shao-Horn, *ACS Energy Lett.* **2024**, *9*, 4883–4891.

[11] H. Kwon, H. Kim, J. Hwang, W. Oh, Y. Roh, D. Shin, H. T. Kim, *Nat. Energy* **2024**, *9*, 57–69.

[12] T. Hou, K. D. Fong, J. Wang, K. A. Persson, *Chem. Sci.* **2021**, *12*, 14740–14751.

[13] L. Xia, S. Lee, Y. Jiang, Y. Xia, G. Z. Chen, Z. Liu, *ACS Omega* **2017**, *2*, 8741–8750.

[14] A. Šimek, T. Kazda, J. Báňa, O. Čech, *Monatshefte fur Chemie* **2024**, *155*, 313–317.

[15] Y. Surace, D. Leanza, M. Mirolo, Ł. Kondracki, C. A. F. Vaz, M. El Kazzi, P. Novák, S. Trabesinger, *Energy Storage Mater.* **2022**, *44*, 156–167.

[16] P. Wardman, *J. Phys. Chem. Ref. Data* **1989**, *18*, 1637–1755.
